# Handwriting in Mild Cognitive Impairment: Reliability Assessment and Machine Learning–Based Screening

**DOI:** 10.2196/73074

**Published:** 2025-09-23

**Authors:** Simone Toffoli, Carlo Abbate, Francesca Lunardini, Edoardo Corno, Nicholas Diani, Alessia Gallucci, Emanuele Tomasini, Pietro Davide Trimarchi, Simona Ferrante

**Affiliations:** 1 Department of Electronics, Information and Bioengineering Politecnico di Milano Milan Italy; 2 IRCCS Fondazione Don Carlo Gnocchi ONLUS Milan Italy; 3 Center for Clinical Neuroscience, Hospital Los Madroños Madrid Spain; 4 LEARNLab, Joint Research Platform, IRCCS Istituto Neurologico Carlo Besta Milan Italy

**Keywords:** mild cognitive impairment, handwriting, sensorized ink pen, machine learning, parole-non-parole test

## Abstract

**Background:**

Mild cognitive impairment (MCI) is a precursor of dementia. Therefore, MCI identification and monitoring are crucial to delaying dementia onset. Given the limits of existing clinical tests, objective support tools are needed.

**Objective:**

This work investigates quantitative handwriting analysis, tailored to enable domestic monitoring, as a noninvasive approach for MCI screening and assessment.

**Methods:**

A sensorized ink pen, used on paper and equipped with sensors, memory, and a communication unit, was used for data acquisition. The tasks included writing a grocery list and free text to mimic daily life handwriting, and a clinical dictation test (parole-non-parole [PnP] test), featuring regular, irregular, and made-up words, aimed at assessing MCI dysgraphia. From the recorded data, 106 indicators describing the performance in terms of time, fluency, exerted force, and pen inclination were computed. A total of 57 patients with MCI were recruited, of whom 45 performed a test-retest protocol. The indicators were examined to assess their test-retest reliability. The indicators from the test repetition were used to assess their relationship with the scores of clinical tests via correlation analysis. For the PnP test, differences in the indicators among the 3 types of words were statistically investigated. These analyses were conducted separately for the cursive (2/3 of the sample) and block letters (1/3 of the sample) allographs, with the level of significance set at 5%. Data from healthy older adults were available for the grocery list (34 participants) and free text (45 participants) tasks. These were exploited to build machine learning classification models for the distinction between patients with MCI and healthy controls.

**Results:**

When dealing with reliability, 93% and 44% of the indicators were characterized by a significant reliability of at least moderate intensity for cursive and block letters respectively. As for the correlation analysis, patients with preserved cognitive status and daily life functionality were associated with significantly better temporal performances, both in free writing and PnP. The analysis of PnP highlighted the presence of surface dysgraphia in the recruited sample, as irregular words showed significantly worse temporal indicators with respect to regular and made-up ones. The classification models’ built-in free writing data achieved accuracies ranging from 0.80 to 0.93 and *F*_1_-scores from 0.81 to 0.92 according to the input dataset.

**Conclusions:**

The presented results suggest the suitability of ecological handwriting analysis for the all-around monitoring of MCI, from early screening to disease progression evaluation.

## Introduction

In the last decades, the world has witnessed progressive aging. This trend brings about various challenges at the social, economic, and health care levels, as it directly affects citizens’ health, compromising several aspects of their daily lives [1]. First, older individuals experience greater difficulty in responding to external stimuli, a condition that leads to isolation and inertia. Second, a gradual, albeit sometimes slow, decline is observed, causing a state referred to as “frailty” [2]. In this state, the individual becomes more susceptible to motor-related accidents, resulting in disability and an increase in hospitalization. Furthermore, aging is linked to a higher prevalence of neurodegenerative diseases, for which treatments that slow down the progression of the disease are the only available interventions.

Among these disorders, dementia is one of the most prevalent, being characterized by a decline in cognitive function, memory loss, and changes in behavior [3]. Dementia leads to a gradual loss in the ability to perform daily tasks independently, causing patients to often require round-the-clock care and supervision. This can translate into emotional and physical toll on family members and caregivers, who may experience stress, burnout, and financial strain. The health care systems are not immune to the negative consequences of the disorder, since it is associated with extensive long-term care, medications, and support services [4].

Clinically, a transitional phase between the healthy state and dementia has been identified and denoted as mild cognitive impairment (MCI) [1]. MCI can manifest heterogeneously. While patients with amnestic MCI experience memory-related cognitive loss, participants diagnosed with nonamnestic MCI face impairments in the domains of attention, language, and visuospatial ability [5]. From an epidemiological standpoint, the aggregate prevalence of MCI among older people ranges from 15% to 20% [6-9]. The problem lies in the relevant yearly percentage (up to 15%) of cases that are diagnosed with dementia after having previously been in the MCI state. This underscores the need for MCI early screening, aimed at preventing the progression to dementia [10]. Unfortunately, the broad spectrum of symptoms and the typically nonsignificant impact on the participants’ independent life make the detection of MCI quite challenging.

Cognitive tests are typically used in clinical practice for this purpose since they are quicker and cheaper than imaging techniques [11,12]. The existing tests, all conducted under the clinician’s supervision, vary in the assessed cognitive domains (eg, memory, language, attention) and in the tasks proposed to the individual, but are in general capable of providing a snapshot of one’s cognitive status. The most common are the Mini-Mental State Examination (MMSE) [12], the Montreal Cognitive Assessment (MoCA) [13], and the clock drawing test (CDT) [14]. Despite being largely used, such clinical tests experience a few drawbacks. As they depend on the clinician administering the test, interrater reliability and subjectivity in the assessment are a concern. Moreover, to assess all the relevant cognitive domains, a combination of different tests should be considered, thus leading to increased administration time. Finally, the scales are usually characterized by low granularity, thus making it difficult to establish universally accepted cutoff scores. Thus, there is a need for a cheap, noninvasive, objective solution to complement the standard clinical procedure for MCI screening [6].

In this sense, the recent literature claimed the importance of handwriting: given its complexity, it can serve as a biomarker for cognitive decline [15]. This is because handwriting is the result of the interaction between various cognitive processes: attention, language, short and long-term memory, motor planning, visual-spatial skills, in-hand manipulation, fine motor control, and sensory awareness of the fingers [16]. With the goal of characterizing the handwriting gesture of patients with MCI or with dementia with respect to healthy participants, several studies have been conducted. In Werner et al [17], a group of 41 controls, 31 patients with MCI, and 22 patients with mild dementia were recruited and performed copying tasks of increasing complexity on a digitizer. Significant differences emerged, with both groups of patients showing increased execution times and reduced pressure during the motor task. Conversely, the Livescribe Echo Pen, used on the Livescribe paper sheet, was used in Kawa et al [16] to compare the handwriting of 37 controls and 37 patients diagnosed with MCI. Again, writing slowness emerged as a peculiar characteristic of the latter group. This was coupled with significantly higher and wider written traces. The extracted features were then used to build a linear discriminant classifier, achieving an accuracy of 70%. The combination of a specific digital pen and paper was also adopted in [18], investigating Chinese handwriting in patients affected by Alzheimer disease, for which MCI is a common precursor. The results confirmed the existing literature, as worse temporal measures, both on paper and in the air, were associated with the disease. A higher degree of pressure applied to the writing surface and a poorer control of the same were revealed as well. A classification study, aimed at distinguishing patients in the early stage of dementia from healthy participants, was conducted in [19]. Cursive loops were acquired with a WACOM digitizer, to then apply unsupervised clustering followed by a Bayesian-based classifier. The approach reached an accuracy of 74%. Execution speed, kinematics complexity, acceleration, and pressure were computed from the data recorded by a WACOM digital graphic tablet in [20]. Handwriting tasks, ranging from drawings to spontaneous sentence production, were performed on a paper sheet fixed onto the tablet surface by a group of 17 healthy participants (healthy control [HC]), 12 patients with MCI, and 23 patients with dementia (AD). Performance results ranged from 69.2% (HC vs MCI vs AD) to 96.6% (HC vs MCI). Pressure features were the most influential in the model output. In Chai et al [21], simple graphic tasks, going from repeatedly writing the letter “T” to drawing a pentagram, were performed by 39 HCs and 40 patients with MCI on a commercial tablet using a digital pen. After extracting 8 features, a binary support vector classifier achieved an *F*_1_-score of 83.1%.

Given the evidence that emerged in the literature, this work aims to evaluate handwriting in patients with MCI by changing the paradigm in terms of the acquisition tool and the proposed handwriting tasks, with an eye on the feasibility of remote, ecological monitoring. Indeed, while able to provide relevant, objective data on the handwriting performance, limitations can be pointed out for the approaches found in the literature. Digitizers can still represent a barrier for older adults [22], who are typically the ones affected by MCI. Moreover, the writing execution on the digitizer surface, characterized by a reduced friction with respect to paper, is inevitably altered [23]. On the other hand, the methods proposed in studies by Kawa et al [16] and Qi et al [18] rely on the combination of a digital device and writing surface, potentially hampering their everyday employment. For these reasons, in this study, handwriting data were collected with a sensorized ink pen [24] that writes on normal paper. As for the tasks, the “Parole-non-Parole” test (PnP), aimed at assessing the origin of dysgraphic manifestations associated with MCI, was administered together with 2 unconstrained exercises, aimed at mimicking the natural handwriting one could perform in the domestic scenario. From the collected data, a series of indicators was extracted, and 3 specific objectives were investigated. First, the test-retest reliability of the indicators was assessed, both for the clinical PnP test and the 2 ecological tasks, to evaluate whether the proposed approach could be suitable for the longitudinal monitoring of handwriting in patients with MCI. Second, the indicators were examined in terms of their quantitative support for the clinical evaluation. This was done by assessing the indicators’ correlation with the scores of clinical tests. Moreover, the PnP was specifically analyzed to understand whether the indicators can be used to support the identification of lexical and phonological dysgraphia. Last, the unconstrained handwriting tasks were considered to build classification models to distinguish between patients with MCI and healthy participants, with the aim of enabling the ecological, domestic monitoring of handwriting as a screening tool for the first signs of cognitive decline.

## Methods

### Ethical Considerations

The protocol received the approval (ID 07_20/05/2021) from the Ethical Committee of the section IRCCS Fondazione Don Carlo Gnocchi of the Ethical Committee IRCCS Regione Lombardia. An information sheet for study participation was provided to all eligible participants, and informed consent was obtained from each of them.

### Participants and Protocol

Participants were recruited by IRCCS Fondazione Don Carlo Gnocchi Milano. The inclusion criteria were (1) confirmed diagnosis of MCI according to the recommendations by Albert et al [25]; (2) being aged older than 65 years; (3) having completed elementary school; (4) having a Clinical Dementia Rating (CDR) scale [26] score less than or equal to 1; (5) obtaining negative neurological examination results for both lateral and extrapyramidal signs; (6) having trail making test A (TMT-A) results within the normal range for age and education according to the Italian calibration by Giovagnoli et al [27]; and (7) not having motor limitations that hinder task performance. Candidates were excluded in case of the presence of cognitive impairment of other origin (cerebral stroke, multiple sclerosis, and Parkinson disease), psychiatric disorders in either recent or remote anamnesis, severe sensory or intellectual deficits, and history of substance abuse or alcohol misuse. The recruited patients underwent clinical assessment, including MMSE, CDR, CDT, TMT-A, basic activity of daily living (B-ADL) [28], and instrumental activity of daily living (I-ADL) [29].

The sample size was determined by taking the assessment of the extracted indicators’ test-retest reliability as the main aim, since the scientific literature lacks similar works on patients with MCI. The only comparable study pertains to a systematic review that reports the psychometric properties of motor assessments (mobility, walking, balance, and functional performance) for patients with dementia, yielding intraclass correlation coefficients (ICC) between 0.42 and 0.99 [30]. On the other hand, the reliability of the proposed sensorized pen was demonstrated, the results showing 0.68<ICC<0.99, only in older adults without any diagnoses [24]. Given the scarcity of specific evidence in the population with MCI, a conservative approach was selected. Considering the minimum ICC value of 0.42 as a target, a statistical power of 80% and a dropout rate of 10%, the sample size was set to 40.

The protocol was administered under the clinician’s supervision in a test-retest fashion on the same day, with at least a 30-minute interval between the 2 repetitions (test and retest) of the protocol. The time interval was chosen to ensure that the participants were under the same conditions between test and retest. In this sense, potential learning effects were not a concern given the nature of the protocol (no memory-related tasks were administered). Each patient was provided with blank sheets of paper to perform three writing tasks in random order, 1 minute apart: (1) writing a grocery list of at least 6 items (List); (2) composing a content-free text of at most 7 lines (Text); and (3) executing the PnP test [31], where the participant wrote a series of 15 words under dictation. These included 7 regular words (simple and easily understandable Italian words: “tavolo,” “cartone,” “attore,” “sorella,” “postini,” “cuoceva,” and “democratiche”), 3 irregular words (requiring proper semantic and lexical comprehension of the Italian language to be written correctly: “cieco,” “nacquero,” and “conoscenza”), and 5 made-up (MU) words (words that do not exist in the Italian dictionary: “sabomi,” “fule,” “descia,” “sterpanzi,” and “getrunna”). The order in which words were presented was fixed across participants and protocol repetitions. Orthographic errors were then evaluated to assess the eventual presence of lexical dysgraphia, which is associated with errors in irregular words, or phonological dysgraphia, linked with errors in MU words.

### Sensorized Ink Pen

The handwriting exercises were carried out using a sensorized ink pen [24], able to record linear acceleration and angular velocity on 3 orthogonal axes, together with the force applied on the pen tip with a sampling frequency of 50 Hz. The device was successfully used for the quantitative analysis of drawing tasks in patients with Parkinson disease [32] and demonstrated to be capable of recognizing healthy, older adults of different ages from unconstrained handwriting samples [33]. Data acquisition was controlled by the operator administering the protocol through a custom iOS app. On top of managing the start and stop of the recordings, the app allows users to store 4 different types of labels upon pressing buttons displayed on the user interface. Labels were used to tag the 3 types of words of the PnP test, so that they could be successively analyzed.

### Data Analysis

The data recorded by the sensorized ink pen were elaborated in MATLAB R2022b (MathWorks, Inc) to extract 106 indicators measuring heterogeneous characteristics of the handwriting gesture, namely the temporal performance, the force patterns applied on the writing surface, the movement fluency, the pen inclination with respect to the vertical axis (tilt), the high frequency oscillations both in the domains of time and frequency. The complete description of the indicators can be found in [32-35]. The indicators presented in the Results section are described in Textbox 1 for each domain. The details on their computation can be found in the Multimedia Appendix 1. The ones with (S) after the name were selected a priori for the analysis of reliability, relationships with clinical scores, and MCI dysgraphia characterization. If all the indicators within a domain were selected a priori, (S) is found at the end of the name of the domain.

**Description of the indicators extracted from the sensorized ink pen data, divided by domain.**

**Temporal domain (S):**

- Execution time: the time in seconds required to complete the execution.
- Rel stroke num: the number of strokes (tracts on paper) generated in the time unit.
- Mean on sheet: the average time in seconds required to produce a stroke.
- On sheet CV: the coefficient of variation (CV) of the time required to produce a stroke.
- On sheet ratio: the percentage of time spent with the tip in contact with the paper with respect to the total time required to complete the execution.
- Mean in air: the average time in seconds spent with the pen in the air.
- In air CV: the CV of the time spent with the pen in the air.
- Mean pause: the average duration in seconds of pauses (in air moments longer than 2 seconds).
- Pause num: the number of pauses in the execution.
- Air sheet ratio: the ratio between the mean in air and the mean on the sheet.

**Fluency domain:**

- LDLJ A (S): the logarithmic dimensionless jerk of the acceleration signal.
- LDLJ G (S): the logarithmic dimensionless jerk of the angular velocity signal.
- SPARC (S): the spectral arc length of the angular velocity signal.
- G NC (S): the number of changes (NC) in the time unit in the angular velocity signal.
- A NC: the number of changes (NC) in the time unit in the acceleration signal.
- Cons peak diff G (S): the absolute average difference in deg/s between consecutive extrema in the angular velocity signal.

**Force domain (S):**

- Mean force: the mean force in arbitrary units exerted while the tip is on paper.
- Force overshoot: the difference between the maximum and median exerted force, in arbitrary units.
- Force NC: the NC in the time unit in the force signal.
- Cons peak diff F: the absolute average difference in arbitrary units between consecutive extrema in the force signal.

**Pen inclination domain:**

- Mean tilt (S): the average pen inclination with respect to gravity in degrees.
- Tilt CV (S): the CV of the pen inclination with respect to gravity.
- Tilt median bandwidth: the width of the frequency interval that contains 50% of the tilt power spectrum total power.

**High frequency oscillations (time domain):**

- A ApEn: approximate entropy of the acceleration signal.

**High frequency oscillations (frequency domain):**

- G peak power: the power at the angular velocity power spectrum peak.
- G RPW 8: the median relative power of the angular velocity power spectrum in a 3 Hz interval centered around 8 Hz.
- G RPW 11: the median relative power of the angular velocity power spectrum in a 3 Hz interval centered around 11 Hz.
- G Dom RPW 11: the relative power of the dominant angular velocity power spectrum in a 3 Hz interval centered around 11 Hz
- A peak frequency: the frequency at which the peak in the acceleration power spectrum is found.
- A peak power: the power at the acceleration power spectrum peak.
- A median bandwidth: the width of the frequency interval that contains 50% of the acceleration power spectrum total power.
- A Dom 68% Peak Bandwidth: the width of the frequency interval, centered around the spectral peak, that contains 68% of the acceleration power spectrum total power.

### Statistical Analysis

#### Overview

The same software was used to run the statistical analyses, with the significance level set at 5%. Given the differences in the indicators in cursive and block letters (BL) handwriting—the choice of the allograph was left free to the participant—the analyses were conducted separately for the 2 allographs. The pipeline was common for each goal: after testing for the indicator normality with the Lilliefors test, the proper statistical test was applied.

#### Reliability

The indicators test-retest reliability in the MCI group for the List, Text, and PnP tasks, as well as for PnP regular, irregular, and MU words separately, was the first aspect to be evaluated. Before computing the reliability, a paired sample test was conducted, namely the *t* test for normally distributed indicators, the Wilcoxon test otherwise. If the paired test rejected the null hypothesis (ie, the mean/median of the difference between test and retest value is equal to 0), the indicator was deemed not reliable. Otherwise, the ICC (absolute agreement) or the Kendall W was computed if the indicator was normal or nonnormal, respectively. Both parameters range between 0 and 1. The indicator reliability was interpreted as follows: nonreliable if ICC<0.5 or W<0.2; fairly reliable if 0.2≤W<0.4; moderately reliable if 0.5≤ICC< 0.75 or 0.4≤W<0.6; good reliability if 0.75≤ICC<0.9 or 0.6≤W<0.8; optimal reliability if ICC≥0.9 or W≥0.8. Notably, the test-retest reliability analysis did not involve the indicators measuring variability (namely, all the indicators measuring variance or coefficient of variation [CV]).

The successive statistical analyses were solely based on the indicators extracted from the first repetition of the task, to avoid including eventual learning phenomenon in the data.

#### Relationship With Clinical Scores

For the second aim (ie, support to the clinical evaluation), the correlation between the indicators and the clinical parameters (MMSE, CDT, B-ADL, I-ADL, TMT-A, and number of errors in PnP test, either globally and divided by word type) was computed to measure to which extent the former can be used to approximate the results of cognitive/functional tests. Either the Pearson (*r*, normal) or Spearman (*ρ*, nonnormal) correlation coefficients were considered with the following interpretation: weak correlation, 0.2≤∣*r*, *ρ*∣<0.4; moderate correlation, 0.4≤∣*r*, *ρ*∣<0.6; strong correlation, 0.6≤∣*r*, *ρ*∣<0.8; very strong correlation, ∣*r*, *ρ*∣≥0.8. In this case, the PnP task was considered as a whole (ie, no differentiation among word types).

#### MCI Dysgraphia Characterization

To provide quantitative information for the detection of lexical and phonetical dysgraphia, handwriting indicators were statistically compared among the different types of words in the PnP task. After averaging the indicators across words of the same type, the differences among the 3 types were studied using statistical methods for paired samples: repeated measures analysis of variance for normal data and Friedman test for the nonnormal case. Following such tests, in case the null hypothesis was rejected, a post hoc comparison was conducted to determine which word groups exhibited significant differences using the Bonferroni method.

### Classification of Unconstrained Handwriting Tasks

Last, binary classification models for the distinction between patients diagnosed with MCI and HC participants were developed in Python (version 3.10; Python Software Foundation). The models were based only on the unconstrained writing tasks presented in this study (List and Text) to collect evidence on the suitability of ecological handwriting for MCI detection. The data for HC participants were collected during other acquisition campaigns, using the same device. These participants signed an informed consent for their voluntary participation. It is worth noting that only participants with age ≥65 years and MMSE ≥27 were included in the HC group, to avoid considering participants with possibly relevant cognitive deterioration [36]. The age threshold of 65 years was based on the age of the youngest participant in the MCI group. Importantly, HCs were excluded if diagnosed with any neurological, musculoskeletal, or cardiovascular conditions that could impair handwriting.

The starting datasets were as follows: (1) List in cursive allograph, L (C); (2) Text in cursive allograph, T (C); (3) List and Text in cursive allograph, L+T (C); (4) List in BL allograph, L (BL); (5) Text in BL allograph, T (BL); (6) List in all allographs (L); and (7) Text in all allographs (T). Independent of the considered dataset, the model’s input consisted of handwriting indicators only, excluding the ones showing a correlation coefficient greater than 0.9 in absolute value with at least another indicator [37]. A 10-fold stratified repeated cross-validation approach was adopted in the training phase, setting the *F*_1_-score as the metric to be optimized, given that, in general, the datasets were not balanced. At each iteration, the seed was changed and the whole dataset was divided into train, validation, and test sets, adopting 0.70, 0.15, and 0.15 ratios, respectively. The validation set was exploited to optimize the algorithm hyperparameters through a randomized search approach running 30 iterations. The test set was used to evaluate the model’s performance on data not seen by the algorithm in the previous phases. The global performances were then assessed by averaging the classification metrics (accuracy, recall, precision, *F*_1_-score, and area under the precision recall curve) over the 10 folds. Within this framework, several strategies were adopted. Given the general imbalance of the target classes, both undersampling and oversampling techniques were implemented in the training and validation sets through the *imbalanced-learn* library [38]. The former included random undersampling, the NearMiss method, and the Estimated Nearest Neighbors method. As for the latter, Synthetic Minority Over-sampling Technique (SMOTE), SMOTE-support vector machine (SVMSMOTE), Borderline SMOTE, and ADASYN were considered. Last, the selectKBest feature selection method [39] was used to reduce the dataset dimensionality, with the number of features K to be tried ranging from 10 to 50 with discrete steps of 10. Support vector classifier, random forest, and a pool of boosting algorithms—namely AdaBoost, gradient boosting classifier (GBC), XGBoost (XGB), LightGBM (LGBM), and CatBoost—were tried. The details on the space of explored hyperparameters for each model are provided in the Multimedia Appendix 1. After the identification of the best performing models on the test set, the importance of indicators in their predictions was evaluated through Shapley Additive Explanations (SHAP) analysis [40]. The results of the SHAP analysis were used to qualitatively explore the patterns of misclassified samples: for each model, the trend of the 5 most relevant indicators for the predictions in misclassified samples was compared with the median trend of the same indicators in the MCI and HC groups through polar plots. To do so, the indicators were normalized between 0 and 1 for visualization purposes.

## Results

### Participants and Dataset

The characteristics of the recruited participants are reported in Table 1, separately for the whole sample of patients with MCI, which was exploited for the classification analysis, and for the sample that performed test and retest acquisitions, considered for the statistical analyses. Among the 57 patients with MCI enrolled, 40 were amnestic and 17 were nonamnestic. In more detail, the sample included: 3 pure amnestic MCIs (with isolated memory disturbance); 37 multiple-domain amnestic MCIs (with memory disturbance associated with disturbance of other cognitive faculties); 8 multiple-domain nonamnestic MCIs (with disturbance of other cognitive faculties, eg, attention, executive, spatial, and language, without memory disturbance); 9 single-domain nonamnestic MCIs (with disturbance of a single cognitive faculty, mostly dysexecutive).

As for the HC sample, information is displayed separately for the List and Text tasks. For this group, only the MMSE score was available. When comparing the MMSE score of participants with MCI and HC, the score of the MCI whole group was significantly lower than that of the HC sample who performed the List (*P*=5.4×10^–05^) and the HC sample who performed the Text (*P*=6.4×10^–07^).

The time required for completing the PnP test, given in seconds as median (IQR), was 126.42 (102.56-155.27) seconds at test and 122.56 (95.16-141.47) seconds at retest.

The allographs adopted by patients who underwent the test-retest protocol are reported hereafter:

- List, 29 cursive, 12 BL, 4 mixed (ie, BL in one repetition and cursive in the other).
- Text, 33 cursive, 11 BL, 1 mixed.
- PnP, 31 cursive, 11 BL, 3 mixed.

The allograph distribution of the data used in the classification problem is shown in Table 2. Here, the information refers to the available handwriting data, since both the test and retest acquisitions of the MCI group were considered, while HC participants performed the tasks multiple times.

**Table 1:** Sample characteristics.

Characteristics			MCI^a^ (whole; N=57)		MCI (test-retest; n=45)	HC^b^ (list; n=34)		HC (text; n=45)
**Sex**								
	Female	32		30		18	23	
	Male	16		15		16	22	
	NA^c^	9		—^d^		—	—	
Age (years), mean (SD)			78.92 (5.59)		78.22 (5.15)	73.35 (7.79)		73.44 (7.50)
Education, median (IQR)			10 (8-13)		12 (8-13)	8 (5-13.5)		8 (5-15)
MMSE^e^, median (IQR)			27 (24-28)		27 (24-28)	29 (28-30)		29 (28-30)
CDR^f^, median (IQR)			0.5 (0.5-0.5)		0.5 (0.5-0.5)	—		—
CDT^g^, median (IQR)			3 (3-5)		3 (3-5)	—		—
B-ADL^h^, median (IQR)			6 (5-6)		6 (5-6)	—		—
I-ADL^i^, median (IQR)			6 (4-8)		6 (4-8)	—		—
TMT-A^j^, median (IQR)			64 (48-89)		67 (51-90)	—		—
PnP^k^ errors, median (IQR)			4 (2-5)		4 (2-5)	—		—
PnP errors R^l^, median (IQR)			1 (0-1)		1 (0-1)	—		—
PnP errors I^m^, median (IQR)			1 (0-1)		1 (0-1)	—		—
PnP errors MU^n^, median (IQR)			2 (1-3)		2 (1-3)	—		—

^a^MCI: mild cognitive impairment.

^b^HC: healthy control.

^c^NA: not available.

^d^Not applicable.

^e^MMSE: Mini-Mental State Examination.

^f^CDR: clinical dementia rating.

^g^CDT: clock drawing test.

^h^B-ADL: basic activity of daily living.

^i^I-ADL: instrumental activity of daily living.

^j^TMT-A: trail making test–A.

^k^PnP: parole-non-parole test.

^l^R: regular words.

^m^I: irregular words.

^n^MU: made-up words.

**Table 2:** Allograph distribution in the datasets used for the binary classification problem.

Task			Cursive	Block letters	NA^a^
**MCI^b^**					
	List	62		28	20
	Text	68		21	3
	List and text	61		21	0
**HC^c^**					
	List	26		21	5
	Text	106		34	5
	List and text	17		6	0

^a^NA: not available.

^b^MCI: mild cognitive impairment.

^c^HC: healthy control.

### Reliability

The analysis regarded the data collected from participants with MCI, denoted as “MCI Test-Retest” in Table 1. Participants who adopted different allographs across the test-retest protocol were excluded. For the sake of brevity, the results are reported for a subset of temporal, force, fluency, and tilt indicators, since these domains are the most commonly investigated in the literature.

Table 3 displays the results obtained on the 2 unconstrained handwriting tasks, separately for cursive (“C” columns) and block letters (“BL” columns) allograph.

In the List executed in cursive, optimal, good, and moderate reliability was revealed for 6 of 15, 8 of 15 (mean pause not reaching significance), and 1 of 15 indicators, respectively. The trend was confirmed in the Text task, with 7, 5, and 2 indicators showing optimal, good, and moderate reliability. Only a single unreliable indicator emerged, namely “Mean Force.” When dealing with BL, the results were comparable for the Text, with only 2 indicators being not reliable. Among the reliable ones, statistical significance was not reached in 4 of 13 cases (mean in air, mean pause, LDLJ A, mean tilt). The situation was instead slightly worse for the List task: reliability was mostly of moderate intensity (10/15 indicators), coupled with 8 cases with a *P* value greater than the critical threshold of .05.

The indicators’ reliability related to the PnP task performed in cursive (Table 4) was optimal, as the analysis yielded 14 out of 15 significant results of at least good reliability. Significance was not revealed only for the mean in air. The breakdown of the 3 types of words (Table 4) revealed that the positive results obtained on the whole task were due to the contribution of regular (Force NC being the only nonreliable indicator) and irregular words (besides cons peak diff G, all the indicators were significantly reliable). MU words were indeed characterized by 5 out of 15 unreliable indicators and 2 nonsignificant results among the reliable ones. Furthermore, in this case, the BL allograph was associated with worse outcomes in the whole PnP (Table 5: 5 unreliable indicators and 6 at least moderately reliable but not significant ones). Here, the poorest results happened with irregular words (6 reliable indicators reaching significance, 6 not reliable), while MU words revealed better reliability values than the corresponding in cursive, although only 4 showed a *P* value lower than .05.

**Figure 1:**
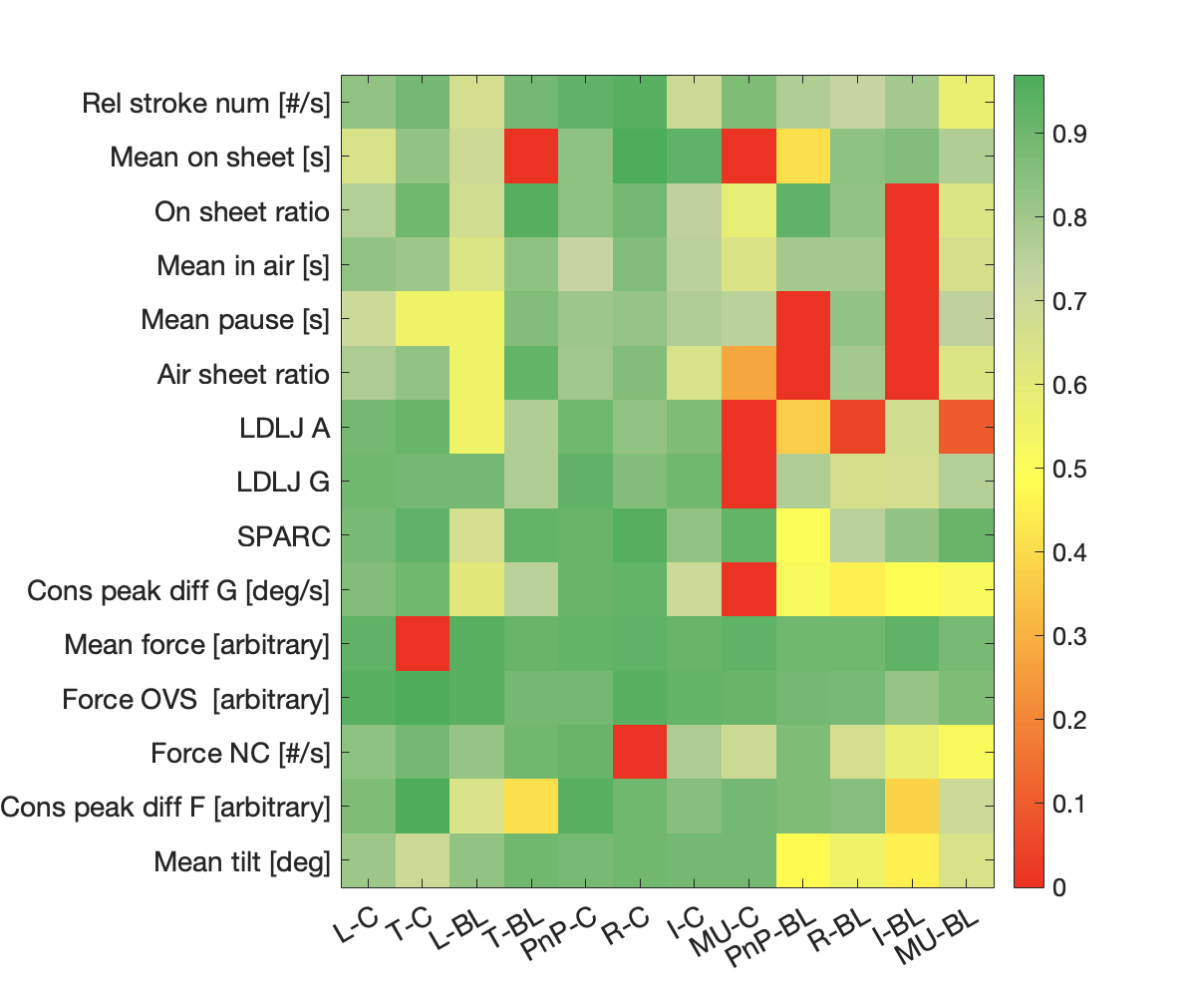
Reliability heatmap. Each row represents an indicator extracted from the sensorized ink pen with its measurement unit (if absent, the indicator is dimensionless), while each column represents a task. The color bar on the right encodes the reliability intensity. For the sake of visualization, here there is no distinct interpretation between ICC and Kendall W for the color coding: only the reliability value is considered. BL: block letters; C: cursive; I: irregular words in PnP; L: list; LDLJ A: logarithmic dimensionless jerk of the acceleration signal; LDLJ G: logarithmic dimensionless jerk of the angular velocity signal; MU: made-up words in PnP; NC: number of changes; OVS: overshoot; PnP: parole-non-parole; R: regular words in PnP; SPARC: spectral arc length of the angular velocity signal; T: text.

Figure 1 displays a heatmap of the overall reliability intensity across indicators—the same reported in Tables 3-5—and tasks.

**Table 3:** Reliability results for List (“L” columns) and Text (“T” columns). Refer to Textbox 1 for descriptions of the indicators.

Domain and indicator			L (C)^a^							T (C)							L (BL)^b^							T (BL)				
			N^c^		Rel^d^		*P* value		N			Rel		*P* value		N			Rel		*P* value		N			Rel		*P* value
**Temporal domain**																												
	Rel stroke num (number/s)	0		0.83^e^		1.8×10^–5^		0			0.89^e^		8.2×10^–9^		0			0.67^f^		.046		0			0.89^e^		.001	
	Mean on sheet (s)	0		0.65^f^		.005		1			0.83^g^		.01		0			0.69^f^		.03		0			0.00^h^		.74	
	On sheet ratio	0		0.76^e^		3.0×10^–4^		0			0.90^g^		8.5×10^–10^		0			0.68^f^		.04		0			0.95^g^		1.7×10^–5^	
	Mean in air (s)	1		0.83^g^		.02		0			0.81^e^		7.5×10^–6^		1			0.63^f^		.24		1			0.84^g^		.08	
	Mean pause (s)	1		0.70^e^		.09		0			0.55^f^		.02		1			0.55^f^		.36		1			0.86^g^		.07	
	Air sheet ratio	1		0.78^e^		.03		0			0.83^e^		2.3×10^–6^		0			0.55^f^		.12		1			0.92^g^		.05	
**Fluency domain**																												
	LDLJ A	0		0.89^e^		8.3×10^–8^		1			0.91^g^		.003		0			0.55^f^		.11		1			0.77^e^		.12	
	LDLJ G	1		0.90^g^		.007		0			0.89^e^		6.4×10^–9^		1			0.89^g^		.05		0			0.77^e^		.01	
	SPARC	0		0.88^e^		2.9×10^–7^		0			0.93^g^		1.3×10^–11^		0			0.67^f^		.04		1			0.92^g^		.05	
	Cons peak diff G (deg/s)	1		0.86^e^		.01		1			0.90^g^		.004		0			0.61^f^		.08		0			0.75^e^		.02	
**Force domain**																												
	Mean force (arbitrary)	0		0.93^g^		2.4×10^–9^		0			NA^h,i^		NA		0			0.94^g^		2.2×10^–5^		0			0.91^g^		.001	
	Force OVS (arbitrary)	1		0.94^g^		.005		0			0.97^g^		1.1×10^–16^		0			0.94^g^		3.0×10^–5^		0			0.89^e^		.001	
	Force NC (number/s)	0		0.8^e^		4.4×10^–6^		0			0.89^e^		4.4×10^–9^		0			0.82^e^		.003		0			0.90^g^		3.3×10^–4^	
	Cons peak diff F (arbitrary)	1		0.87^g^		.01		0			0.97^g^		4.4×10^–16^		0			0.65^f^		.06		0			0.41^h^		.20	
**Tilt domain**																												
	Mean tilt (deg)	1		0.81^g^		.02		0			0.69^f^		8.7×10^–4^		1			0.83^g^		.08		1			0.90^g^		.05	

^a^C: cursive.

^b^BL: block letters.

^c^N: normality (0 normal, 1 nonnormal).

^d^Rel: reliability.

^e^Good reliability.

^f^Moderate reliability.

^g^Excellent reliability.

^h^Not reliable.

^i^Indicator failed the paired samples test.

**Table 4:** Reliability results for parole-non-parole test (PnP) test and regular (R), irregular (I), and made-up (MU) words in cursive. Refer to Textbox 1 for descriptions of the indicators.

Domain and indicator			PnP (C)^a^							R							I							MU				
			N^b^		Rel^c^		*P* value		N			Rel		*P* value		N			Rel		*P* value		N			Rel		*P* value
**Temporal domain**																												
	Rel stroke num (number/s)	1		0.93^d^		.003		1			0.94^d^		.003		0			0.69^e^		.001		1			0.87^d^		.008	
	Mean on sheet (s)	1		0.84^d^		.01		1			0.97^d^		.002		1			0.93^d^		.003		1			NA^f,g^		NA	
	On sheet ratio	0		0.84^h^		2.5×10^–5^		0			0.89^d^		2.8×10^–8^		1			0.74^h^		.049		1			0.59^e^		.24	
	Mean in air (s)	1		0.72^h^		.06		0			0.86^h^		2.8×10^–7^		1			0.75^h^		.04		1			0.64^h^		.14	
	Mean pause (s)	1		0.81^d^		.02		1			0.82^d^		.02		1			0.77^h^		.03		1			0.75^d^		.04	
	Air sheet ratio	1		0.80^d^		.02		0			0.86^h^		3.2×10^–7^		0			0.65^e^		.004		0			0.28^g^		.20	
**Fluency domain**																												
	LDLJ A	1		0.90^d^		.005		0			0.83^h^		3.6×10^–6^		0			0.87^h^		2.2×10^–7^		1			NA^g^		NA	
	LDLJ G	1		0.93^d^		.003		1			0.86^d^		.01		0			0.90^d^		6.3×10^–9^		1			NA^g^		NA	
	SPARC	1		0.91^d^		.004		1			0.95^d^		.002		1			0.83^d^		.014		1			0.92^d^		.004	
	Cons peak diff G (deg/s)	1		0.91^d^		.004		1			0.92^d^		.004		1			0.70^h^		.077		1			NA^g^		NA	
**Force domain**																												
	Mean force (arbitrary)	0		0.92^d^		8.1×10^–10^		0			0.93^d^		1×10^–10^		0			0.91^d^		3.6×10^–9^		0			0.93^d^		2.9×10^–10^	
	Force OVS (arbitrary)	0		0.89^h^		2.8×10^–8^		0			0.94^d^		4.2×10^–11^		1			0.92^d^		.004		1			0.91^d^		.004	
	Force NC (number/s)	0		0.91^d^		4.9×10^–9^		0			NA^g^		NA		0			0.78^h^		4.2×10^–5^		0			0.69^e^		.001	
	Cons peak diff F (arbitrary)	0		0.94^d^		4.3×10^–12^		0			0.90^d^		6.4×10^–9^		0			0.85^h^		8.6×10^–7^		1			0.89^d^		.006	
**Tilt domain**																												
	Mean tilt (deg)	0		0.88^h^		4.4×10^–8^		0			0.90^d^		8.3×10^–19^		0			0.89^h^		6.2×10^–8^		0			0.89^h^		4.8×10^–8^	

^a^C: cursive.

^b^N: normality (0 normal, 1 nonnormal).

^c^Rel: reliability.

^d^Excellent reliability.

^e^Moderate reliability.

^f^Indicator failed the paired samples test.

^g^Not reliable.

^h^Good reliability.

**Table 5:** Reliability results for parole-non-parole test (PnP) test and regular (R), irregular (I), and made-up (MU) words in block letters. Refer to Textbox 1 for descriptions of the indicators.

Domain and indicator		PnP (BL)^a^			R			I			MU		
		N^b^	Rel^c^	*P* value	N	Rel	*P* value	N	Rel	*P* value	N	Rel	*P* value
**Temporal domain**													
	Rel stroke num (number/s)	0	0.77^d^	.01	1	0.72^d^	.15	0	0.79^d^	.007	0	0.57^e^	.10
	Mean on sheet (s)	0	0.41^f^	.17	0	0.84^d^	.003	0	0.86^d^	.002	0	0.77^d^	.01
	On sheet ratio	0	0.93^g^	3.4×10^–4^	1	0.83^g^	.09	0	NA^f,h^	NA	1	0.63^d^	.25
	Mean in air (s)	1	0.79^d^	.11	0	0.79^d^	.01	1	NA^f^	NA	0	0.66^e^	.06
	Mean pause (s)	0	NA^f^	NA	1	0.83^g^	.09	1	NA^f^	NA	1	0.74^d^	.14
	Air sheet ratio	0	NA^f^	NA	0	0.79^d^	.009	1	NA^f^	NA	0	0.63^e^	.07
**Fluency domain**													
	LDLJ A	0	0.37^f^	.24	0	0.04^f^	.47	0	0.68^d^	.04	0	0.10^f^	.44
	LDLJ G	1	0.77^d^	.12	0	0.66^e^	.06	0	0.67^d^	.03	0	0.76^d^	.02
	SPARC	0	0.50^e^	.15	0	0.75^d^	.02	1	0.83^g^	.08	1	0.91^g^	.05
	Cons peak diff G (deg/s)	0	0.51^e^	.15	0	0.45^d^	.20	0	0.48^f^	.16	0	0.51^e^	.15
**Force domain**													
	Mean force (arbitrary)	1	0.90^g^	.05	1	0.90^g^	.05	1	0.93^g^	.05	1	0.88^g^	.06
	Force OVS (arbitrary)	1	0.89^g^	.06	0	0.88^d^	.001	0	0.82^d^	.004	1	0.87^g^	.07
	Force NC (number/s)	0	0.87^d^	.001	0	0.67^e^	.06	1	0.58^e^	.32	0	0.51^e^	.12
	Cons peak diff F (arbitrary)	0	0.87^d^	.001	0	0.85	.003	0	0.38^f^	.25	0	0.70^e^	.04
**Tilt domain**													
	Mean tilt (deg)	0	0.41^f^	.17	0	0.55^e^	.08	1	0.45^i^	.52	0	0.65^e^	.04

^a^BL: block letters.

^b^N: normality (0 normal, 1 nonnormal).

^c^Rel: reliability.

^d^Good reliability.

^e^Moderate reliability.

^f^Not reliable.

^g^Excellent reliability.

^h^Indicator failed the paired samples test.

^i^Fair reliability.

### Relationship With Clinical Scores

The statistically significant correlations of handwriting indicators with clinical parameters are reported in Table 6. Overall, significant correlations were identified mainly for the unconstrained tasks in BL allograph. The I-ADL score was also significantly correlated with the indicators extracted from the Text in cursive allograph, while the cursive List exhibited no significant results at all. The PnP test, on the other hand, revealed relationships with the computed indicators mainly when considering the number of errors in the irregular words. The correlation absolute values ranged from moderate to strong, and their direction followed the expected trends.

**Table 6:** Correlation results. The correlation coefficient value and its corresponding *P* value are reported for all the tasks, separately for cursive (C) and block letters (BL) allographs. The clinical parameter “PnP errors - I” refers to the number of errors with irregular words in the parole-non-parole test (PnP) test. The correlations, in this case, were computed only with the indicators extracted from irregular words. Refer to Textbox 1 for descriptions of the indicators.

Clinical parameter and indicator			L^a^ (C)^b^				T^c^ (C)				L (BL)^d^				T (BL)				PnP (C)				PnP (BL)		
			corr^e^		*P* value		corr		*P* value		corr		*P* value		Corr	*P* value		corr			*P* value		corr		*P* value
**MMSE^f^**																									
	Rel stroke num (number/s)	—^g^		—		—		—		0.52		.04		0.66		.00	—			—		—		—	
	On sheet ratio	—		—		0.41		.03		0.62		.01		0.57		.02	—			—		0.51		.04	
	Mean in air (s)	—		—		—		—		–0.56		.02		–0.66		.00	—			—		—		—	
	Mean pause (s)	—		—		—		—		—		—		–0.63		.01	—			—		–0.51		.05	
	Pause num (number)	—		—		–0.42		.02		–0.68		.004		—		—	—			—		–0.74		.001	
	Air sheet ratio	—		—		—		—		–0.58		.02		–0.57		.02	—			—		—		—	
**CDR^h^**																									
	On sheet CV	—		—		—		—		—		—		–0.80		.047	–0.80			.047		—		—	
	Mean in air (s)	—		—		0.80		.047		—		—		0.80		.047	—			—		—		—	
	Air sheet ratio	—		—		—		—		—		—		0.80		.047	—			—		—		—	
**CDT^i^**																									
	Mean force (arbitrary)	—		—		—		—		0.50		.05		—		—	—			—		0.53		.03	
	Force OVS (arbitrary)	—		—		—		—		0.59		.02		0.58		.02	—			—		—		—	
	Force NC (number/s)	—		—		—		—		0.63		.01		—		—	—			—		—		—	
	Cons peak diff F (arbitrary)	—		—		—		—		0.57		.02		—		—	—			—		0.74		.001	
**I-ADL^j^**																									
	Rel stroke num (number/s)	—		—		0.42		.02		0.57		.02		0.65		.01	—			—		—		—	
	Mean on sheet (s)	—		—		–0.42		.02		—		—		—		—	—			—		—		—	
	On sheet ratio	—		—		—		—		0.66		.01		0.65		.01	—			—		—		—	
	Mean in air (s)	—		—		–0.49		.01		–0.63		.01		–0.83		.00	—			—		—		—	
	Mean pause (s)	—		—		–0.41		.03		—		—		–0.52		.04	—			—		—		—	
	Pause num (number)	—		—		–0.48		.01		–0.57		.02		—		—	—			—		–0.69		.003	
	Air sheet ratio	—		—		—		—		–0.61		.01		–0.73		.001	—			—		—		—	
**TMT-A^k^**																									
	Mean in air (s)	—		—		0.47		.01		—		—		0.55		.03	—			—		—		—	
	Mean force (arbitrary)	—		—		—		—		—		—		–0.51		.04	—			—		—		—	
	Force NC (number/s)	—		—		–0.39		.04		–0.51		.045		—		—	–0.41			.03		—		—	
	Cons peak diff F [arbitrary]	—		—		—		—		–0.58		.02		–0.59		.02	—			—		—		—	
**PnP errors – I**																									
	Mean on sheet (s)	—		—		—		—		—		—		—		—	—			—		–0.65		.01	
	On sheet ratio	—		—		—		—		—		—		—		—	—			—		–0.69		.007	
	Air sheet ratio	—		—		—		—		—		—		—		—	—			—		0.70		.005	
	G NC (number/s)	—		—		—		—		—		—		—		—	–0.54			.002		–0.61		.02	

^a^L: list.

^b^C: cursive.

^c^T: text.

^d^BL: block letters.

^e^corr: correlation.

^f^MMSE: Mini-Mental State Examination.

^g^Not applicable.

^h^CDR: clinical dementia rating.

^i^CDT: clock drawing test.

^j^I-ADL: instrumental activity of daily living.

^k^TMT-A: trail making test-A.

### MCI Dysgraphia Characterization

The statistically significant results of the post hoc comparison of indicators (ie, the indicators for which the repeated measures ANOVA or Friedman test rejected the null hypothesis) among regular, irregular, and MU words are reported in Table 7 for both cursive (“C” columns) and block letters (“BL” columns). Most significances regarded irregular words.

**Table 7:** Results of the parole-non-parole test post-hoc comparison among different word types. “Type 1” and “Type 2” indicate the type of words being compared. The columns “LB,” “Diff,” and “UB” represent, respectively, the lower bound, the estimate, and the upper bound of the difference in the indicator between the type 1 and type 2 words. Refer to Textbox 1 for descriptions of the indicators.

Indicator	Type 1	Type 2	C^a^						BL^b^				
			*P* value^c^	LB^d^	Diff	UB^e^	*P* value (PH)	*P* value		LB	Diff	UB	*P* value (PH)^f^
**Execution time (s)**													
	R^g^	I^h^	1.7×10^–6^	–1.52	–0.90	–0.28	.001	3.3×10^–4^		–2.05	–1.14	–0.24	.007
	MU^i^	I	1.7×10^–6^	–1.92	–1.30	–0.68	1.4×10^–6^	3.3×10^–4^		–2.33	–1.43	–0.52	4.7×10^–4^
**Rel stroke num (number/s)**													
	R	I	4.4×10^–5^	0.42	1.03	1.65	1.9×10^–4^	.025		0.07	0.35	0.63	.015
	MU	I	4.4×10^–5^	0.35	0.97	1.58	5.4×10^–4^	.025		0.07	0.36	0.66	.015
**On sheet ratio**													
	R	I	.88	—^j^	—	—	—	1.6×10^–4^		0.03	0.10	0.17	.005
	MU	I	.88	—	—	—	—	1.6×10^–4^		0.03	0.12	0.22	.007
**Mean in air (s)**													
	R	I	5.9×10^–5^	–1.28	–0.67	–0.05	.029	3.4×10^–4^		–2.33	–1.43	–0.52	4.7×10^–4^
	MU	I	5.9×10^–5^	–1.75	–1.13	–0.52	3.4×10^–5^	3.4×10^–4^		–2.05	–1.14	0.24	.007
**In air CV**													
	R	MU	.001	0.06	0.22	0.38	.004	.008					
	MU	I	.001	–0.33	–0.17	0.00	.044	.008		0.05	–0.32	0.59	.019
**Mean pause (s)**													
	R	I	1.5×10^–5^	–1.39	–0.82	–0.24	.002	.002		–1.84	–1.04	–0.23	.006
	MU	I	1.5×10^–5^	–1.66	–1.08	–0.51	1.8×10^–5^	.002		0.20	–1.00	1.80	.009
**Pause num (number)**													
	MU	I	.002	–0.92	–0.55	–0.18	.001	.018		–1.20	–0.64	–0.01	.016
**Tilt CV**													
	R	I	.003	0.25	0.87	1.48	.002	6.3×10^–4^		0.52	1.43	2.33	4.7×10^–4^
	MU	I	.003	—	—	—	—	6.3×10^–4^		0.02	0.93	1.83	.042
**LDLJ G**													
	R	MU	.032	–1.28	–0.67	–0.05	.029	.001		–0.39	–0.22	–0.04	.013
	MU	I	.032	—	—	—	—	.001		0.11	0.34	0.57	.004

^a^C: cursive.

^b^BL: block letters.

^c^*P* value of the statistical test investigating the differences among the 3 types of words (ANOVA or Friedman test).

^d^UB: upper bound.

^e^LB: lower bound.

^f^PH: *P* value of the pairwise post hoc comparison.

^g^R: regular.

^h^I: irregular.

^i^MU: made-up.

^j^Not applicable.

### Classification of Unconstrained Handwriting Tasks

The best classification performances for each dataset are displayed in Table 8, together with the adopted training strategy in terms of under- or oversampling and feature selection. Classification metrics on the test set are given as average (SD) over the 10 folds of stratified repeated cross-validation.

**Table 8:** Binary classification performances on the test set. For details, see the Methods section (Data Analysis subsection).

Dataset	Model	Sampling^a^	FS^b^	Accuracy	Recall	Precision	*F*_1_-score	PRC^c^ AUC^d^
L^e^ (C)^f^	RF^g^	SMOTE^h^	1	0.84 (0.05)	0.93 (0.07)	0.83 (0.08)	0.87 (0.04)	0.82 (0.07)
T^i^ (C)	CB^j^	SMOTE	0	0.86 (0.06)	0.87 (0.08)	0.81 (0.14)	0.83 (0.07)	0.76 (0.12)
L+T (C)	AB^k^	No	0	0.84 (0.09)	0.96 (0.08)	0.85 (0.09)	0.90 (0.07)	0.84 (0.09)
L (BL)^l^	CB	SVMSMOTE^m^	1	0.80 (0.14)	0.90 (0.17)	0.78 (0.18)	0.81 (0.13)	0.77 (0.17)
T (BL)	SVC^n^	RUS^o^	0	0.93 (0.07)	1.00 (0.00)	0.87 (0.14)	0.92 (0.09)	0.87 (0.14)
L	XGB^p^	SMOTE	0	0.84 (0.07)	0.88 (0.07)	0.88 (0.09)	0.87 (0.06)	0.85 (0.09)
T	SVC	RUS	0	0.86 (0.08)	0.85 (0.12)	0.79 (0.12)	0.81 (0.10)	0.73 (0.13)

^a^The adopted sampling strategy is reported.

^b^FS is 1 if feature selection was applied, 0 otherwise.

^c^PRC: precision recall curve.

^d^AUC: area under the curve.

^e^L: list.

^f^C: cursive.

^g^RF: random forest.

^h^SMOTE: Synthetic Minority Oversampling Technique.

^i^T: text.

^j^CB: CatBoost.

^k^AB: AdaBoost.

^l^BL: block letters.

^m^SVMSMOTE: Synthetic Minority Oversampling Technique-Support Vector Machine.

^n^SVC: support vector classifier.

^o^RUS: random undersampling.

^p^XGB: XGBoost.

The top 5 most important indicators for each model, according to the SHAP analysis, are presented in Table 9.

A total of 21 indicators resulted among the possible 35 (5 top relevant indicators for 7 datasets), the most relevant ones. The most prevalent was Tilt CV, being present in 5 out of 7 datasets (one occurrence of Tilt CV*_T_* in the L+T [C] dataset), followed by G peak power (4/7). The indicators A peak frequency and force NC were found in 3/7 cases, while cons peak diff G, A ApEn, and execution time resulted in 2/7 datasets. The remaining indicators occurred only once. Importantly, the indicators occurring multiple times were always characterized by the same direction in the MCI group. A high degree of consistency was revealed for the 3 models trained on cursive samples. Indeed, Tilt CV was the most important indicator in all 3 cases, while cons peak diff G, A ApEn, and G peak power were found in the 2 models trained with List cursive samples (L [C]; L+T [C]). For the 2 models trained on BL samples, the opposite trend occurred, with no superpositions among their 5 most relevant indicators.

Given these outcomes, the analysis of misclassified samples was carried out for cursive-samples models only.

In the L (C) model, G peak power showed trends compatible with those of patients with MCI in 50% (5/10) of false positive (FP) samples. The other recurrent altered indicators in FPs were cons peak diff G and air sheet ratio (4/10, 40%). Tilt CV and G peak power were found to be in line with that of HC participants in 83% (5/6) and 67% (4/6) of the false negatives (FNs), respectively.

In T (C), FPs were mainly associated with problems in temporal indicators (execution time: 11/16, 69%; mean pause: 10/16, 63%) and in a peak frequency (11/16, 69%). This last indicator was the main contributor to the presence of FNs (12/16, 75%), together with Tilt CV and mean pause (10/16, 63%).

When combining the information of the 2 tasks (L+T [C]), Tilt CV*_T_* was found to deviate from the expected values in most misclassified samples: 67% in both FPs (4/6) and FNs (2/3). The other main contributor to FPs was A NC (3/6, 50%).

**Table 9:** For each indicator, the indicator name, the corresponding absolute Shapley Additive Explanations value, and the direction in patients with MCI are presented for each dataset. The results are related to the best-performing model in each dataset, presented in Refer to Textbox 1 for descriptions of the indicators.

Dataset	#1 Indicator	#2 Indicator	#3 Indicator	#4 Indicator	#5 Indicator
L^a^ (C)^b^	Tilt CV 0.093 (+)^c^	Cons peak diff G 0.057 (+)	A ApEn 0.044 (+)	Air sheet ratio 0.019 (+)	G peak power 0.019 (+)
T^d^ (C)	Tilt CV 0.129 (+)	Force NC 0.090 (–)	Execution time 0.083 (+)	A_Peak frequency 0.058 (–)	Mean pause 0.042 (+)
L+T (C)	Tilt CV_T_^e^ 0.090 (+)	A ApEn 0.059 (+)	A NC 0.056 (+)	Cons peak diff G 0.036 (+)	G peak power 0.010 (+)
L (BL)^f^	LDLJ A 0.108 (–)^g^	A_Peak frequency 0.105 (–)	G peak power 0.066 (+)	Force overshoot 0.041 (+)	On sheet CV 0.022 (+)
T (BL)	Force NC 0.042 (–)	G RPW 11 0.040 (+)	A Dom 68% peak bandwidth 0.039 (+)	G RPW 8 0.035 (+)	Tilt median bandwidth 0.032 (–)
L	Tilt CV 0.077 (+)	Execution time 0.073 (+)	A peak frequency 0.041 (–)	G peak power 0.027 (+)	G Dom RPW 11 0.024 (+)
T	Force NC 0.063 (–)	A peak power 0.043 (+)	A median bandwidth 0.043 (+)	G NC 0.039 (+)	Tilt CV 0.030 (+)

^a^L: list.

^b^C: cursive.

^c^+ denotes increased indicator values.

^d^T: text.

^e^Tilt CV_T_ in the L+T (C) dataset refers to the Tilt CV indicator in the text task. The other indicators in the row refer to the list task.

^f^BL: block letters.

^g^– denotes decreased indicator values.

Interestingly, 3 HCs and 2 MCIs were misclassified across all 3 cursive datasets. As for the HC group:

- PEN024 was consistently misclassified because of trends in A ApEn and temporal indicators (execution time, mean pause, and air sheet ratio) compatible with those of patients with MCI.
- PEN025 instead showed alterations in the kinematics of the handwriting movement, with high values of Tilt CV in the T (C) task and great angular velocity oscillations power in both tasks (G peak power).
- PENT002 exhibited a heterogeneous pattern, with difficulties in the force and pen inclination domains in the T (C) task. Altered values of cons peak diff G were among the reasons for the wrong prediction both in L (C) and L+T (C).

Moving to the patients with MCI:

- DGSMN07 revealed a high coherence across tasks. The modulation of rotational kinematics (cons peak diff G and G peak power) was similar to that of HCs, in turn causing the same behavior in Tilt CV.
- DGMSN17 was misclassified in all datasets due to the trend of Tilt CV. The effect of temporal indicators was identified in the T (C) task.

**Figure 2:**
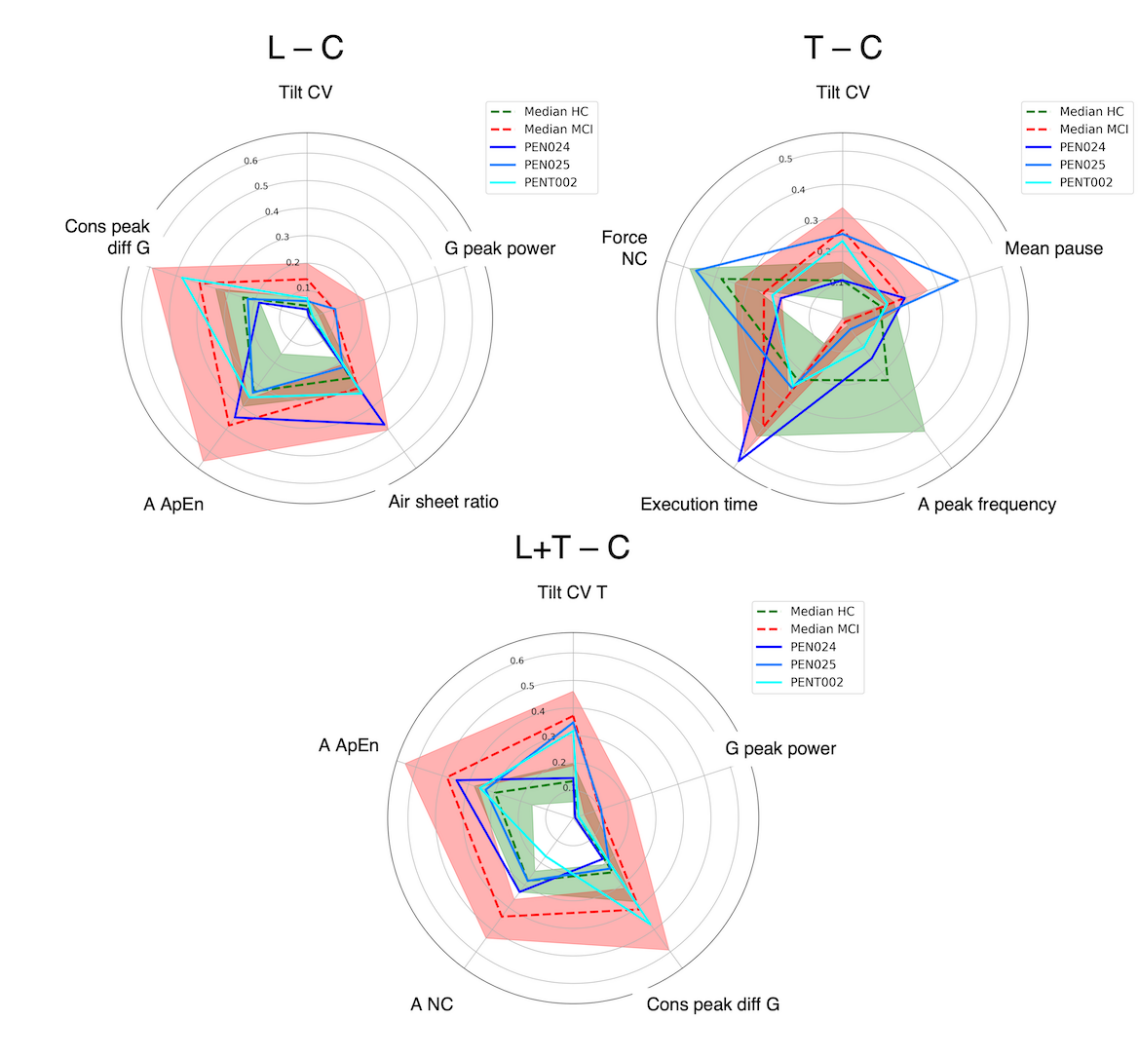
Radar plots for HC samples from participants PEN024, PEN025, and PENT002. A ApEn: approximate entropy of the acceleration signal; C: cursive; CV: coefficient of variation; HC: healthy control; L: list; MCI: mild cognitive impairment; T: text; Tilt CV T: tilt CV indicator in the text task.

**Figure 3:**
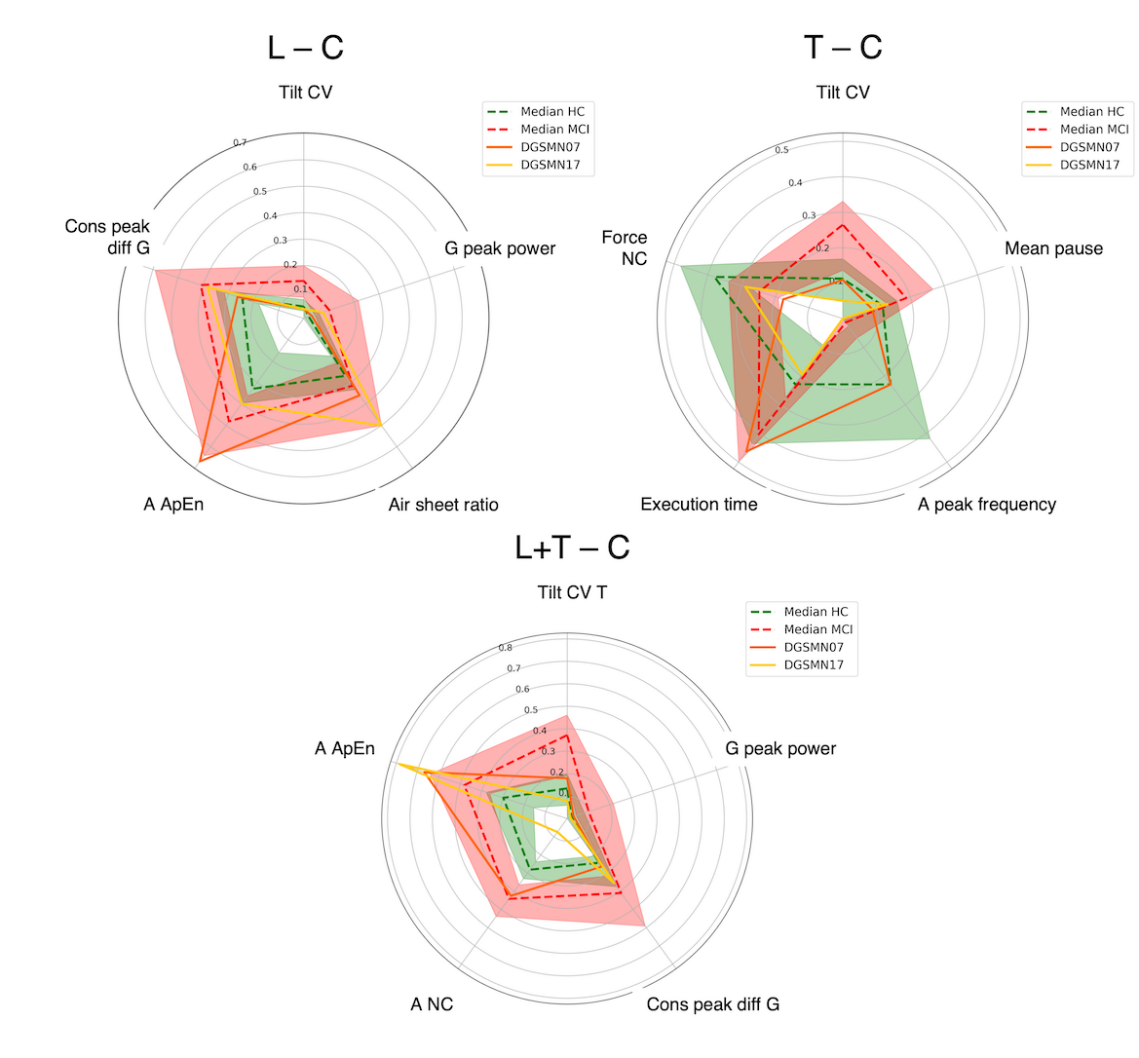
Radar plots for MCI samples from participants DGSMN07 and DGSMN17. A ApEn: approximate entropy of the acceleration signal; C: cursive; CV: coefficient of variation; HC: healthy control; L: list; MCI: mild cognitive impairment; T: text; Tilt CV T: tilt CV indicator in the text task.

The indicators’ trend for these samples is presented in the following radar plots for each classification model (in order, L [C], T [C], and L+T [C]). For each model, the most relevant indicator is displayed at the top of the radar. Then, indicators are displayed counterclockwise in decreasing order of relevance. All the values are normalized between 0 and 1 for visualization purposes. The dashed green line represents the indicators’ median value, while the green area represents the interval between the 25th and the 75th percentile in the HC group. The red elements represent the same for the group of patients with MCI. The solid lines represent the indicator’s value for the sample under examination. Blue hues are used for HC samples in Figure 2, while orange hues are used for MCI samples in Figure 3.

## Discussion

### Principal Findings

This work presented the quantitative analysis of handwriting in a population of patients diagnosed with MCI. With respect to the existing body of literature on the topic, an ecological approach for data acquisition was proposed. Handwriting tasks were executed on traditional paper using a sensorized ink pen able to record kinematic and dynamic signals. Moreover, content-free handwriting composition tasks were proposed to the participants to mirror the writing that can be carried out in everyday life. These aspects were deemed necessary for the development of a solution that potentially unlocks the monitoring of handwriting in the home environment. However, such features alone are not sufficient for the purpose.

That is why the first goal of the work was the assessment of the test-retest reliability of the information extracted from the acquisition protocol. High reliability is an essential requirement for any measurement instrument, particularly for solutions meant to detect anomalies in unsupervised longitudinal frameworks, in order to avoid high FP rates and alert fatigue. Importantly, the reliability analysis was conducted by separating different writing styles, namely cursive and BL, given that they differ in both temporal and kinematic parameters. The results were excellent for the cursive allograph, considering the 3 tasks, 42 out of the 45 presented indicators resulted significantly reliable, with reliability values mostly rated as good or optimal. This percentage dropped for BL: this was caused mainly by *P* values not reaching statistical significance, rather than by low reliability values. The outcome could be explained by the reduced number of participants who adopted the BL allograph in the recruited sample, which could also be the reason behind the overall better results found for cursive. Interestingly, the best results were achieved with the unconstrained tasks (List and Text), regardless of the adopted allograph. This could imply that one’s peculiar handwriting process characteristics are preserved independently of the content itself, thus making the analysis of unsupervised handwriting activity a promising approach for the remote tracking of MCI progression. On the other hand, the reliability of the indicators from the standardized PnP test in BL was not always verified. The unreliability of the mean pause indicator, measuring the average duration of moments longer than 2 seconds spent with the pen in the air, could, in theory, be due to differences in the dictation timing of the operator between test and retest acquisitions.

Regarding the support for the clinical evaluation, the correlation analysis revealed a scarce presence of significant results (around 10% of the total correlation values). Such an outcome should not be interpreted negatively for 2 reasons. First, the administered clinical tests are not meant to assess the handwriting performance specifically. Even the CDT and the TMT-A, which require the patients to draw and trace lines for their completion, are not evaluated according to the graphomotor performance itself, but rather on their accuracy. The same holds for the PnP test: the scoring is based on the orthographic correctness of the written words, while the proposed indicators are aimed at characterizing the handwriting process. Thus, a direct correspondence between the clinical scores and the handwriting indicators does not exist. Moreover, the CDR and B-ADL scores were characterized by low granularity (the former presented with only 2 distinct values, namely 0.5 and 1), resulting in poor significance. Second, and most importantly, the obtained significant correlations were of at least moderate intensity and in line with the existing literature [17]. Diving into detail, the highest number of correlations was found between temporal handwriting indicators and the MMSE and the I-ADL scores. As the MMSE and I-ADL increased, rel stroke num and on on-sheet ratio increased as well, while the mean in air, the mean pause, pause num, and air sheet ratio decreased. Therefore, in the recruited sample, participants who are more cognitively intact and functional in daily life tend to write faster thanks to a reduced degree of hesitation. Another relevant finding concerns the 2 tests involving the use of a pen or pencil. Those who performed well in the CDT and TMT-A applied a greater force on the writing surface, coupled with a higher number of changes in the force signal direction (the opposite signs of the correlation values are due to the opposite interpretation of the tests score). Such a pattern is associated in the literature with the healthy status [19]. Last, significant correlations between indicators and the number of errors in the PnP test emerged only for the errors in Irregular words. Here, when the BL allograph was used, a higher number of errors was associated with a faster performance on the sheet: the mean time spent with the pen on paper (mean on sheet) and the percentage of time spent on paper when writing a word (on sheet ratio) both showed a negative correlation. This could suggest that errors in irregular words occurred because the participants paid reduced attention (hence the increased speed on the sheet) to the words to be written, despite requiring orthographic knowledge to be written correctly. Linking the observed correlation patterns with specific domains of cognitive function is not trivial. The clinical tests used in the correlational study are useful in providing general measures of one’s cognitive functioning or some of its high-level variables, such as processing speed, or measures of the patient’s level of autonomy. In contrast, they are not thought of, nor have they shown particular accuracy and validity in detecting dysfunctions of individual cognitive functions such as memory, attention, executive functions, critical thinking, language, spatial abilities, visual perception, and so on. Any attempt to explain the obtained correlations in terms of individual cognitive domains would therefore be highly speculative and unsupportable with the available data. However, to a first approximation, the correlations between temporal indicators and MMSE and I-ADL scores seem to closely resemble what clinicians call cognitive hesitancy, an aspect of general insecurity in test responses that can be framed in the context of executive function dysfunctionality.

Dealing with the quantitative information derived from the PnP test, another neat trend emerged for irregular words. Independent of the handwriting style and orthographic errors, irregular words posed the greatest challenge to the recruited sample. With respect to the other types of words, irregular ones were consistently and significantly associated with worse in-air temporal performances (ie, longer times spent with the pen in the air), as revealed by mean in air and mean pause. This translated into a significantly slower rate of grapheme production (Rel stroke num) and, consequently, into the longest times required to write the words (execution time). In the BL allograph, alterations were also found in the on-sheet execution, as irregular words exhibited around 10% less time spent with the pen on the writing surface (on-sheet ratio). Last, irregular words were linked to a reduced variability in the pen inclination *(*Tilt CV), possibly suggesting an impaired manipulation of the writing instrument when dealing with this type of word. According to the classical cognitive models of handwriting, the marked alteration of the handwriting process found in the current work for irregular words, which require semantic and lexical knowledge to be written correctly compared with regular and MU words, could underline alterations in the lexical pathways as a result of dysfunctions in the orthographic retrieval process.

Overall, the implemented quantitative indicators seem able to capture relevant aspects of the handwriting gesture that are linked to the participant’s general conditions. On top of that, the indicators can also identify altered patterns that can provide a deeper understanding of the nature of the patient’s handwriting impairments. This stresses once again the suitability of handwriting monitoring in the population with MCI, as impairments in the gesture could be associated with a cognitive or functional decline.

The last evaluated aspect was the ability of the indicators to distinguish unconstrained handwriting samples generated by patients with MCI from those written by older adults without any diagnoses and MMSE ≥ 27. The choice of focusing on the List and Text tasks was motivated by their potential application in the domestic scenario. In such a condition, the information could be gathered in ecological modality without the need for the operator’s supervision. The classification performances were good, with *F*_1_-scores selected as the evaluation criterion given the datasets’ imbalance, ranging from 0.81 to 0.92. Importantly, the results do not appear to be task or allograph-dependent. These results highlight the power of the proposed analysis: the extracted handwriting process indicators do provide relevant information on the participants’ cognitive status, regardless of the writing content and the allograph adopted to realize it. When comparing with the existing literature, the current results are placed between the accuracies of 74% in [19] and 96.6% in [20]. It is, however, worth pointing out that the latter classification performance, related to the execution of a spontaneous sentence, was obtained in different conditions. A digitizer was used, thus providing information also on the written trace, not available in this study, on a narrow sample size (12 patients with MCI and 17 controls). Moreover, the authors reported the participants’ age, which was not balanced between groups, as one of the most discriminative features for the model. Such a variable was not included in the models presented here.

The exploration of the relevance of indicators in the predictions of models provided great insight into the aspects that mostly differentiate the execution of patients with MCI and HC participants. This was true particularly for the cursive allograph, since heterogeneous results were found in the models based on BL samples, likely because of the reduced sample size. In general, the outcome of the SHAP analysis was not the expected one. Despite anticipating temporal indicators to carry the highest discriminative power, reflecting hesitations and greater processing times for patients with MCI, their occurrence among the most relevant was somewhat limited: only 5 out of 35 temporal indicators emerged, never placed in first or second position. The biggest determinant in the classification models was the variability of the pen inclination with respect to the vertical axis (Tilt CV), as it was found among the 5 most relevant indicators in 6 out of 7 models, being in the first place in 4 cases. In the classification models built on the cursive List samples (L [C] and L+T [C]), the relevance of Tilt CV, with high values always steering the classification towards the MCI group, was coherently associated with the presence of other kinematic indicators, both in the fluency and in the frequency domains. Such an outcome could open new perspectives on the evaluation of the handwriting performance of patients with MCI, traditionally focused on execution time, product quality, and orthographic accuracy. Researchers and clinicians should also consider the handwriting process kinematics that underlie the generation of the written trace. The results of the SHAP analysis were the basis for the inspection of misclassification patterns in the cursive-based models. These were homogeneous in FNs, for whom Tilt CV was the main reason why the models predicted them as belonging to the HC group. When this was not the case, the cause was still found in the handwriting kinematics. The situation was variable for FPs. In L (C), three archetypes emerged: (1) the participants who experience alterations in the variability of pen inclination; (2) the participants with excessive amplitude in rotational movements; (3) a heterogeneous group, with difficulties both in kinematics and in-air durations. The same number of patterns showed up in T (C). The first was again characterized by abnormal values of Tilt CV, while the second exhibited the opposite behavior, with no impairments in Tilt CV. The last archetype was the most anticipated one, where increased times were the sole reason for misclassifications. When combining List and Text in cursive, 2 main patterns were highlighted. Once again, the most prevalent had Tilt CV*_T_* as the cause of FPs occurrence, while the other one was the entropy of the acceleration signal. Overall, the analysis revealed the multifaceted nature of the handwriting process in older adults, highlighting the importance of the participant-specific exploration of the model predictions. This would allow one to unveil one’s peculiar pattern of handwriting impairments, thus helping the clinician in identifying the motor and cognitive aspects that need a thorough assessment.

It is important to highlight the limitations found in this work. Regarding the recruitment, depression and anxiety disorders, relevant for various aspects of cognitive motor processes, were not formally measured during patients’ assessment. However, patients with a history of anxiety disorders or depression were excluded from the sample. So, caution should be placed when trying to generalize the presented findings to the population with MCI. As for the inclusion of control participants, their selection was solely based on their MMSE score. While they did not report any neurological diagnoses, they did not undergo any thorough clinical examination aimed at assessing their cognitive status. Thus, the presence of cognitively impaired participants in the control group cannot be excluded. In the statistical analysis of the available data, the percentage of recruited patients who adopted the BL allograph (around 1/3 of the total) was limited with respect to cursive. This translated into a reduced number of statistical significances in the reliability analysis, while the binary classification models trained on BL samples were characterized by greater SDs of the classification metrics across the 10 folds of the stratified repeated cross-validation, with respect to their cursive counterparts. Despite the imbalance in the adopted allograph, we do believe that the performance difference between BL-trained and cursive-trained classification models does not imply a reduced discriminative power of the former writing style. Although additional BL samples are needed to prove this hypothesis, this allograph should be better suited for the binary classification problem based on the proposed tool and indicators. Indeed, being the allograph characterized by a highly standardized motor pattern of stroke generation, the main source of variability between MCI and HC participants should be found in the indicators measuring the in-air performance, which should reflect longer processing times and hesitations in the former group. When dealing with cursive, the participants’ personal handwriting style comes into play, making the classification task more challenging. Just as an example, greater times on-sheet could indicate either slowness of movement or a writing style characterized by longer, interconnected strokes. In general, the choice of the allograph was left free to the participant to guarantee an ecological execution of the task and to align with the PnP test instructions, which do not specify how the participant should write, but the presented findings should be confirmed by increasing the BL sample size. The free choice turned out to be a liability in a limited number of cases, where patients adopted different allographs in the test and retest acquisitions, thus making them unsuitable for the reliability analysis. Given the impossibility of characterizing specific cognitive domains with the battery of clinical tests used in this work, the inclusion of more specific clinical tests for different cognitive domains is planned as a next step to refine the correlation analysis. In this regard, the positive results obtained with the words and nonwords writing test, which is the only instrument investigating a specific cognitive function in the study, indicate that such a research objective seems promising and feasible. With respect to the digitizers, the used sensorized ink pen cannot directly provide information on the trace made on paper. Future work should focus on proper methods for the extraction of product-related handwriting information to reduce this gap, particularly for the cursive allograph. Other promising directions can be found in multimodal approaches for the classification of patients with MCI. For example, the authors in [21] demonstrated that the fusion of handwriting and EEG parameters can improve the classification performance with respect to considering a single data source alone. Of course, the feasibility and user friendliness of the protocol should be carefully designed in such a case. The results also found the basis for additional research: a longitudinal study could be conducted, possibly in an unsupervised scenario. Last, the clinical community would benefit from comparative investigations on the discriminative efficacy of various handwriting-based screening methods. They should also consider their implementation complexity to provide an all-around informed documentation regarding the utility and feasibility of their integration into clinical practice.

### Conclusions

The relevance of handwriting in the population with MCI was confirmed in this work, which described a novel approach for its quantitative characterization. The analyses revealed 2 potential complementary scenarios for its fruitful application. On one side, information related to the participant’s clinical conditions can be quickly and noninvasively extracted, both through the PnP test and unconstrained handwriting. On the other hand, the handwriting tool emerges as a viable solution to track old adults’ conditions in a transparent and ecological way, fostering MCI remote monitoring in a home setting.

## Appendix

Multimedia Appendix 1 Details on indicators computation and hyperparameter tuning.

## Abbreviations

B-ADL: basic activity of daily living
BL: block letters
C: cursive
CDR: clinical dementia rating
CDT: clock drawing test
CV: coefficient of variation
FN: false negative
FP: false positive
HC: healthy control
I-ADL: instrumental activity of daily living
ICC: intraclass correlation coefficient
L: list
MCI: mild cognitive impairment
MMSE: Mini-Mental State Examination
MU: made-up
NC: number of changes
PnP: parole-non-parole test
RF: random forest
SMOTE: Synthetic Minority Oversampling Technique
SHAP: Shapley Additive Explanations
SVC: support vector classifier
SVMSMOTE: Synthetic Minority Oversampling Technique-support vector machine
T: text
TMT-A: trail making test-A
XGB: XGBoost

## References

[1] Anderson ND (2019). State of the science on mild cognitive impairment (MCI). CNS Spectr.

[2] Gray SL, Anderson ML, Hubbard RA, LaCroix A, Crane PK, McCormick W, Bowen JD, McCurry SM, Larson EB (2013). Frailty and incident dementia. J Gerontol A Biol Sci Med Sci.

[3] Prince M (2007). Epidemiology of dementia. Psychiatry.

[4] Bremer P, Cabrera E, Leino-Kilpi H, Lethin C, Saks K, Sutcliffe C, Soto M, Zwakhalen SM, Wübker A, RightTimePlaceCare Consortium (2015). Informal dementia care: consequences for caregivers' health and health care use in 8 European countries. Health Policy.

[5] Roberts R, Knopman DS (2013). Classification and epidemiology of MCI. Clin Geriatr Med.

[6] Pessoa R, Bomfim A, Ferreira B, Chagas M (2019). Diagnostic criteria and prevalence of mild cognitive impairment in older adults living in the community: a systematic review and meta-analysis. Arc Clin Psychiatr.

[7] Chen P, Cai H, Bai W, Su Z, Tang Y, Ungvari GS, Ng CH, Zhang Q, Xiang Y (2023). Correction to: global prevalence of mild cognitive impairment among older adults living in nursing homes: a meta-analysis and systematic review of epidemiological surveys. Transl Psychiatry.

[8] Ritchie K (2004). Mild cognitive impairment: an epidemiological perspective. Dialogues Clin Neurosci.

[9] Gillis C, Mirzaei F, Potashman M, Ikram MA, Maserejian N (2019). The incidence of mild cognitive impairment: a systematic review and data synthesis. Alzheimers Dement (Amst).

[10] Vessio G (2019). Dynamic handwriting analysis for neurodegenerative disease assessment: a literary review. Appl Sci.

[11] O'Caoimh R, Timmons S, Molloy DW (2016). Screening for mild cognitive impairment: comparison of "MCI Specific" screening instruments. J Alzheimers Dis.

[12] Zhuang L, Yang Y, Gao J (2021). Cognitive assessment tools for mild cognitive impairment screening. J Neurol.

[13] Freitas S, Simões MR, Alves L, Santana I (2013). Montreal cognitive assessment: validation study for mild cognitive impairment and alzheimer disease. Alzheimer Dis Assoc Disord.

[14] Breton A, Casey D, Arnaoutoglou NA (2019). Cognitive tests for the detection of mild cognitive impairment (MCI), the prodromal stage of dementia: meta-analysis of diagnostic accuracy studies. Int J Geriatr Psychiatry.

[15] De Stefano C, Fontanella F, Impedovo D, Pirlo G, Scotto di Freca A (2019). Handwriting analysis to support neurodegenerative diseases diagnosis: a review. Pattern Recognit Lett.

[16] Kawa J, Bednorz A, Stępień P, Derejczyk J, Bugdol M (2017). Spatial and dynamical handwriting analysis in mild cognitive impairment. Comput Biol Med.

[17] Werner P, Rosenblum S, Bar-On G, Heinik J, Korczyn A (2006). Handwriting process variables discriminating mild alzheimer's disease and mild cognitive impairment. J Gerontol B Psychol Sci Soc Sci.

[18] Qi H, Zhang R, Wei Z, Zhang C, Wang L, Lang Q, Zhang K, Tian X (2023). A study of auxiliary screening for alzheimer's disease based on handwriting characteristics. Front Aging Neurosci.

[19] Kahindo C, El-Yacoubi MA, Garcia-Salicetti S, Rigaud A, Cristancho-Lacroix V (2018). Characterizing early-stage alzheimer through spatiotemporal dynamics of handwriting. IEEE Signal Process. Lett.

[20] Garre-Olmo J, Faúndez-Zanuy M, López-de-Ipiña K, Calvó-Perxas L, Turró-Garriga O (2017). Kinematic and pressure features of handwriting and drawing: preliminary results between patients with mild cognitive impairment, alzheimer disease and healthy controls. Curr Alzheimer Res.

[21] Chai J, Wu R, Li A, Xue C, Qiang Y, Zhao J, Zhao Q, Yang Q (2023). Classification of mild cognitive impairment based on handwriting dynamics and qEEG. Comput Biol Med.

[22] Fischer SH, David D, Crotty BH, Dierks M, Safran C (2014). Acceptance and use of health information technology by community-dwelling elders. Int J Med Inform.

[23] Gerth S, Klassert A, Dolk T, Fliesser M, Fischer MH, Nottbusch G, Festman J (2016). Is handwriting performance affected by the writing surface? comparing preschoolers', second graders', and adults' writing performance on a tablet vs. paper. Front Psychol.

[24] Lunardini F, Di Febbo D, Malavolti M, Cid M, Serra M, Piccini L, Pedrocchi ALG, Borghese NA, Ferrante S (2021). A smart ink pen for the ecological assessment of age-related changes in writing and tremor features. IEEE Trans Instrum Meas.

[25] Albert MS, DeKosky ST, Dickson D, Dubois B, Feldman HH, Fox NC, Gamst A, Holtzman DM, Jagust WJ, Petersen RC, Snyder PJ, Carrillo MC, Thies B, Phelps CH (2011). The diagnosis of mild cognitive impairment due to alzheimer's disease: recommendations from the national institute on aging-alzheimer's association workgroups on diagnostic guidelines for alzheimer's disease. Alzheimers Dement.

[26] Morris JC (1997). Clinical dementia rating: a reliable and valid diagnostic and staging measure for dementia of the alzheimer type. Int Psychogeriatr.

[27] Giovagnoli AR, Del Pesce M, Mascheroni S, Simoncelli M, Laiacona M, Capitani E (1996). Trail making test: normative values from 287 normal adult controls. Ital J Neurol Sci.

[28] Jefferson AL, Byerly LK, Vanderhill S, Lambe S, Wong S, Ozonoff A, Karlawish JH (2008). Characterization of activities of daily living in individuals with mild cognitive impairment. Am J Geriatr Psychiatry.

[29] Gold DA (2012). An examination of instrumental activities of daily living assessment in older adults and mild cognitive impairment. J Clin Exp Neuropsychol.

[30] Trautwein S, Maurus P, Barisch-Fritz B, Hadzic A, Woll A (2019). Recommended motor assessments based on psychometric properties in individuals with dementia: a systematic review. Eur Rev Aging Phys Act.

[31] Capasso R, Miceli G (2001). Esame Neuropsicologico per l'Afasia: E.N.P.A.

[32] Toffoli S, Lunardini F, Parati M, Gallotta M, De Maria B, Longoni L, Dell'Anna ME, Ferrante S (2023). Spiral drawing analysis with a smart ink pen to identify Parkinson's disease fine motor deficits. Front Neurol.

[33] Lomurno E, Toffoli S, Febbo DD, Matteucci M, Lunardini F, Ferrante S (2025). Age group discrimination via free handwriting indicators. IEEE J Biomed Health Inform.

[34] Toffoli S, Lunardini F, de Isla CG, Ferrante S (2023). AI-based ecological monitoring of handwriting to early detect cognitive decline.

[35] Toffoli S, Lunardini F, Parati M, Gallotta M, Muletti M (2021). Classification of patients with Parkinson’s disease using free handwriting features collected through a smart ink pen. Universidad de Las Palmas de Gran Canaria.

[36] Salis F, Costaggiu D, Mandas A (2023). Mini-mental state examination: optimal cut-off levels for mild and severe cognitive impairment. Geriatrics (Basel).

[37] Ulivi M, Meroni V, Orlandini L, Prandoni L, Rossi N, Peretti GM, Dui LG, Mangiavini L, Ferrante S (2020). Opportunities to improve feasibility, effectiveness and costs associated with a total joint replacements high-volume hospital registry. Comput Biol Med.

[38] (2024). Imbalanced-Learn Documentation.

[39] (2024). Machine learning in python. scikit-learn.

[40] Lundberg S, Lee S (2017). A unified approach to interpreting model predictions. NIPS'17: Proceedings of Proceedings of the 31st International Conference on Neural Information Processing Systems.

