# Supplementary material for: Handwriting in Mild Cognitive Impairment: Reliability Assessment and Machine Learning–Based Screening
Source: JMIR Aging. 2025 Sep 23;8:e73074. doi: 10.2196/73074 (PMC12504891; doi:10.2196/73074)
Supplement: Multimedia Appendix 1 [file aging_v8i1e73074_app1.docx]

## **Indicators Calculation**

This document describes how the indicators described in the Data Analysis Section are computed. We define:

- fs, the sampling frequency of the sensorized ink pen IMU, force sensor and timestamp;
- *t*, the timestamp time series, measured in seconds;
- *F*, the force signal time series, measured in arbitrary unit;
- *A_x_*, *A_y_* and *A_z_* the linear acceleration time series along the x, y and z axis respectively, measured in mm/s^2^;
- *AV_x_*, *AV_y_* and *AV_z_* the angular velocity time series along the x, y and z axis respectively, measured in deg/s.

### **Stroke Segmentation**

Strokes are defined as segments where the sensorized ink pen tip is in contact with the paper. The single acquisition is characterized by an arbitrary number of strokes, depending on the written content and the subject’s personal handwriting style. The instant of beginning and end of each stroke were identified as follows:

$$t_{start}=t_{i} | F\left( t_{i-1} \right)=0\wedge F\left( t_{i} \right)>0; t_{stop}=t_{i} | F\left( t_{i} \right)>0\wedge F\left( t_{i+1} \right)=0$$

The signals associated to the i-th stroke are defined as:

$$t_{stroke i-th}=t\left( t_{start i-th}, t_{stop i-th} \right); F_{stroke i-th}=F\left( t_{start i-th}, t_{stop i-th} \right)$$

$$A_{x,y,z stroke i-th}=A_{x,y,z}\left( t_{start i-th}, t_{stop i-th} \right); {AV}_{x,y,z stroke i-th}={AV}_{x,y,z}\left( t_{start i-th}, t_{stop i-th} \right)$$

We define *S* the number of strokes found in the raw data. We define *IA* the number of in air moments found in the raw data.

### **Temporal domain**

$$\boldsymbol{Execution Time}\left[ \boldsymbol{s} \right]= t_{start,1}-t_{stop,s}$$

$$\boldsymbol{Rel Stroke Num}\left[ \boldsymbol{\#/s} \right]=\frac{S}{Execution Time}$$

$$\boldsymbol{Mean On Sheet}\left[ \boldsymbol{s} \right]=\frac{\sum_{i=1}^{S} t_{stop, i}-t_{start, i}}{S}$$

$$\boldsymbol{On Sheet CV}\left[ \boldsymbol{s} \right]=\frac{Mean On Sheet}{\sqrt{\frac{\sum_{i=1}^{S} \left[ \left( \sum_{i=1}^{S} t_{stop, i}-t_{start i} \right)-Mean On Sheet \right]^{2}}{S}}}$$

$$\boldsymbol{On Sheet Ratio [ ]}=\frac{\sum_{i=1}^{S} t_{stop, i}-t_{start, i}}{Execution Time};$$

$$\boldsymbol{Mean In Air [s]}=\frac{\sum_{i=1}^{S-1} t_{start, i+1}-t_{stop, i}}{S}$$

$$\boldsymbol{In Air CV [s]}=\frac{Mean In Air}{\sqrt{\frac{\sum_{i=1}^{S-1} \left[ \left( \sum_{i=1}^{S} t_{start, i+1}-t_{start, i} \right)-Mean In Air \right]^{2}}{S-1}}}$$

$$\boldsymbol{Mean Pause}\left[ \boldsymbol{s} \right]=\frac{\sum_{i=1}^{S-1} {(t}_{start, i+1}-t_{stop, i}) |{(t}_{start, i+1}-t_{stop, i})> 2}{IA \left| {(t}_{start}-t_{stop} \right)>2}$$

$$\boldsymbol{Pause Num}\left[ \boldsymbol{\#} \right]=IA \left| {(t}_{start}-t_{stop} \right)>2$$

$$\boldsymbol{Air SheetRatio []} =\frac{Mean In Air}{Mean On Sheet}$$

### **Fluency Domain**

We define *jerk_a_* the derivative of *A*. The index *i* goes from 1 to *S*.

$$\boldsymbol{LDLJ A []}={median}_{x,y,x}({mean}_{S}(-\log\left( \frac{t_{stop, i}-t_{start, i}}{{max(|A_{i}- \frac{\sum_{t_{start,i}}^{t_{stop,i}} {jerk}_{a,i}}{t_{stop, i}-t_{start, i}}|)}^{2}}*\sum_{t_{start,i}}^{t_{stop,i}} {{(jerk}_{a,i})}^{2} \right)))$$

We define *jerk_av_* the derivative of *AV*. The index *i* goes from 1 to *S*.

$$\boldsymbol{LDLJ G []}={median}_{x,y,x}({mean}_{S}(-\log\left( \frac{{(t_{stop, i}-t_{start, i})}^{3}}{{max(|{AV}_{i}|)}^{2}}*\sum_{t_{start,i}}^{t_{stop,i}} {{(jerk}_{av,i})}^{2} \right)$$

We define *AV_FN_* the fourier transform of *AV* normalized for its DC component, *th* the normalized amplitude threshold, *f_cut-off_* the last frequency at which *AV_FN_* is greater than or equal to *th*. The measures is computed for *th = 0.3.*

$$\boldsymbol{S}\boldsymbol{PARC}_{\boldsymbol{th}}[]= -\sum_{i=0}^{f_{cut-off}} \sqrt{\left( \frac{1}{f_{cut-off}-1} \right)^{2}+{\dot{{AV}_{FN}}}^{2}}$$

The angular velocity axis characterized by the highest peak in the power spectrum was selected (*G_dom_*). The number of *G_dom_* maxima and minima was summed up within each stroke to obtain *G NC_i_*, to then average it over *S*:

$$\boldsymbol{G NC [\#/s]}=\frac{\sum_{i=1}^{S} \frac{{G NC}_{i}}{t_{stop i}-t_{start i}}}{S}$$

The 3D acceleration was computed. The number of *A_3D_* maxima and minima was summed up within each stroke to obtain *A NC_i_*, to then average it over *S*:

$$A_{3D}=\sqrt{\left( A_{x}+A_{y}+A_{z} \right)^{2}}; \boldsymbol{A NC [\#/s]}=\frac{\sum_{i=1}^{S} \frac{{G NA}_{i}}{t_{stop i}-t_{start i}}}{S}$$

We define *G_ext_* the angular velocity local extrema within the *i-th* stroke.

$$\boldsymbol{Cons Peak Diff G [deg/s]}={mean}_{S}\left( \frac{\sum_{t=1}^{\#G_{ext} in stroke i} \left| G_{ext,t}-G_{ext,t-1} \right|}{\#G_{ext} in stroke i} \right)$$

### **Force Domain**

The mean force applied in each stroke was computed as:

$$\boldsymbol{Mean Force [arbitrary]}=\frac{\sum_{i=1}^{S} \frac{\sum_{t_{start i}}^{t_{stop i}} F}{t_{stop i}-t_{start i}}}{S}$$

The number of *F* maxima and minima was summed up within each stroke to obtain *F NC_i_*, to then average it over *S*:

$$\boldsymbol{Force NC [\#]}=\frac{\sum_{i=1}^{S} \frac{{F NC}_{i}}{t_{stop i}-t_{start i}}}{S}$$

$$\boldsymbol{Force Overshoot}\left[ \boldsymbol{arbitrary} \right]=\frac{\sum_{i=1}^{S} (\max\left( F_{i} \right)-median(F_{i}))}{S}$$

We define *F_ext_* the force local extrema within the *i-th* stroke.

$$\boldsymbol{Cons Peak Diff F [arbitrary]}={mean}_{S}\left( \frac{\sum_{t=1}^{\#F_{ext} in stroke i} \left| F_{ext,t}-F_{ext,t-1} \right|}{\#F_{ext} in stroke i} \right)$$

### **Pen Inclination Domain**

For Tilt computation, the following steps were followed for each stroke:

1. *A_z_* (the linear acceleration component directed as the longitudinal axis of the pen) was low pass filterd with cut off frequency set to 10Hz *.*
2. A first approximation was computed as: $\theta_{approx}=\sin^{-1} \frac{A_{z stroke i}}{9.81}$
3. Defined constant $k_{1}=1.5$ and $k_{2}=0.4$, the Tilt was updated using the angular velocity tilt estimation as:

$$\theta\left( t \right)=\theta\left( t-1 \right)+k_{2}*\left( {AV}_{x, stroke i} \left( t-1 \right)+{AV}_{y,stroke i}\left( t-1 \right) \right)+k_{1}*\left( \theta_{approx}\left( t-1 \right)-\theta\left( t-1 \right) \right)$$

4. Finally, the following Tilt features were extracted:

$$\boldsymbol{Mean Tilt}\left[ \boldsymbol{deg} \right]= \frac{\sum_{i=1}^{S} \theta_{i}}{S};$$

$$\boldsymbol{Tilt CV[]}= \frac{\sqrt{\frac{1}{S-1}*\sum_{i=1}^{S} |{\theta_{i}-Mean Tilt|}^{2}}}{Mean Tilt}$$

### **High Frequency Oscillations – Time domain**

Firstly, the signals were divided into windows (*W*) of 500 samples each, without distinguishing between on-sheet and in-air tracts. In case of *A_3D_*:

We define *Nw* the number of windows found in the raw data.

The approximate entropy was computed, after removing the frequency content below 2Hz, on each *W_i_* using the MATLAB® routine *approximateEntropy*:

$${ApEn}_{i}=approximateEntropy\left( W_{i},'Dimension^{'}, 2,'Radius^{'}, 0.2*std(W_{i}) \right)$$

Finally, we obtained:

$$\boldsymbol{A ApEn []}=\frac{\sum_{i=1}^{Nw} {ApEn}_{i}}{Nw}$$

### **High Frequency Oscillations – Frequency domain**

We define *PSD_G dom_* the power spectral density (PSD) of *G_dom_*, computed with the welch method (500 samples, 50% overlap), and *f_peak, G dom_* the frequency at which the peak of *PSD_G dom_* is found.

$$\boldsymbol{G Peak Power [}\left( \boldsymbol{deg/s} \right)^{\boldsymbol{2}}\boldsymbol{/Hz]}={PSD}_{G dom}\left( f_{peak, G dom} \right)$$

We define *PSD_G_* the PSD of the angular velocity signal, computed with the welch method (500 samples, 50% overlap).

We define *A_dom_* the acceleration axis characterized by the highest peak in the power spectrum. We define *PSD_A dom_* the power spectral density (PSD) of *A_dom_*, computed with the welch method (500 samples, 50% overlap), and *f_peak, A dom_* the frequency at which the peak of *PSD_A dom_* is found.

$$\boldsymbol{A Peak Frequency [Hz]}=f_{peak, A dom}$$

$$\boldsymbol{A Peak Power [}\left( \boldsymbol{mm/}\boldsymbol{s}^{\boldsymbol{2}} \right)^{\boldsymbol{2}}\boldsymbol{/Hz]}={PSD}_{A dom}\left( f_{peak, A dom} \right)$$

$$\boldsymbol{A Dom 68\% PeakBandwidth}\left[ \boldsymbol{Hz} \right]={(f}_{2}-f_{1}), f_{1},f_{2} | \frac{\sum_{{f=f}_{1}}^{f_{2}} {PSD}_{A}\left( f \right)}{\sum_{f=2 Hz}^{12.5 Hz} {PSD}_{A}\left( f \right)}=0.68$$

We define *PSD_A_* the PSD of the acceleration signal, computed with the welch method (500 samples, 50% overlap).

$$\boldsymbol{A Median Bandwidth}\left[ \boldsymbol{Hz} \right]={median}_{x,y,z}\left( f_{MBW} | \frac{\sum_{f=2 Hz}^{f_{MBW}} {PSD}_{A}\left( f \right)}{\sum_{f=2 Hz}^{12.5 Hz} {PSD}_{A}\left( f \right)}=0.5 \right)$$

We define *PSD_T_* the PSD of the tilt signal, computed with the welch method (500 samples, 50% overlap).

$$\boldsymbol{Tilt Median Bandwidth}\left[ \boldsymbol{Hz} \right]=f_{MBW} | \frac{\sum_{f=2 Hz}^{f_{MBW}} {PSD}_{T}\left( f \right)}{\sum_{f=2 Hz}^{12.5 Hz} {PSD}_{T}\left( f \right)}=0.5$$

## **Hyperparameters tuning**

The space of hyperparameters explored during the hyperparameters tuning via Randomized Search (30 iterations) in the validation phase of the binary classification pipeline is detailed hereafter for each classification algorithm. The names of the hyperparameters refer to their Python® 3.10 implementation. The libraries used were: sklearn, xgboost, lgb and catboost.

### **SVC**

{'kernel':['linear', 'rbf', 'poly'],

'C':[0.1, 1, 10, 100],

'gamma':[0.001,0.01,0.1,1]}

### **Random Forest**

{'n_estimators': [10, 50 ,100],

'criterion': ['gini', 'entropy', 'log_loss'],

'min_samples_split': [2, 5, 10],

'min_samples_leaf': [1, 2, 5],

'max_features': ['sqrt', 'log2', None]}

### **AdaBoost**

{'n_estimators': [10, 50 ,100],

'learning_rate': [0.01,0.03,0.1],

'algorithm': ['SAMME', 'SAMME.R']}

### **Gradient Boosting Classifier**

{'n_estimators': [10, 50],

'learning_rate': [0.01,0.1],

'subsample': [0.5, 0.75, 1],

'min_samples_split': [2, 5],

'min_samples_leaf': [2, 5],

'max_depth': [None, 3, 5],

'n_iter_no_change': [None, 20]}

### **XGBoost**

{'n_estimators': [50 ,100, 150],

'eta': [0.1, 0.3, 0.5],

'gamma': [0, 1, 5],

'max_depth': [3, 5, 8],

'min_child_weight': [1, 2, 3],

'subsample': [0.5, 0.75, 1]}

### **LigthGBM**

{'n_estimators': [50 ,100, 150],

'learning_rate': [0.1, 0.3, 0.5],

'num_leaves': [30, 40, 50],

'min_split_gain': [0, 1, 5],

'min_child_weight': [0.001, 0.01, 0.1],

'subsample' : [0.5, 0.75, 1]}

### **Catboost**

{'depth': [2,4,6,8],

'learning_rate': [0.01, 0.03, 0.1],

'iterations': [250, 500, 1000]}
